# A Probiotic Mixture Induces Anxiolytic- and Antidepressive-Like Effects in Fischer and Maternally Deprived Long Evans Rats

**DOI:** 10.3389/fnbeh.2020.581296

**Published:** 2020-11-12

**Authors:** Valérie Daugé, Catherine Philippe, Mahendra Mariadassou, Olivier Rué, Jean-Charles Martin, Marie-Noelle Rossignol, Nathalie Dourmap, Ljubica Svilar, Franck Tourniaire, Magali Monnoye, Deborah Jardet, Marie Bangratz, Sophie Holowacz, Sylvie Rabot, Laurent Naudon

**Affiliations:** ^1^Université Paris-Saclay, INRAE, AgroParisTech, CNRS, Micalis Institute, Jouy-en-Josas, France; ^2^Université Paris-Saclay, INRAE, AgroParisTech, Micalis Institute, Jouy-en-Josas, France; ^3^Université Paris-Saclay, INRAE, MaIAGE, Jouy-en-Josas, France; ^4^Université Aix Marseille, C2VN, CRIBIOM, Marseille, France; ^5^Université Paris-Saclay, INRAE, BioinfOmics, MIGALE Bioinformatics Facility, Jouy-en-Josas, France; ^6^UNIROUEN, UFR Médecine-Pharmacie, Inserm U 1245 Team 4, Rouen, France; ^7^Université Aix Marseille, C2VN, Marseille, France; ^8^PiLeJe Laboratoire, Paris, France

**Keywords:** probiotic, maternal deprivation, anxiety-like behavior, depressive-like behavior, monoamines, brain, metabolites, gut junction protein RNAs

## Abstract

A role of the gut microbiota in psychiatric disorders is supported by a growing body of literature. The effects of a probiotic mixture of four bacterial strains were studied in two models of anxiety and depression, naturally stress-sensitive Fischer rats and Long Evans rats subjected to maternal deprivation. Rats chronically received either the probiotic mixture (1.10^9^ CFU/day) or the vehicle. Anxiety- and depressive-like behaviors were evaluated in several tests. Brain monoamine levels and gut RNA expression of tight junction proteins (Tjp) and inflammatory markers were quantified. The gut microbiota was analyzed in feces by 16S rRNA gene sequencing. Untargeted metabolite analysis reflecting primary metabolism was performed in the cecal content and in serum. Fischer rats treated with the probiotic mixture manifested a decrease in anxiety-like behaviors, in the immobility time in the forced swimming test, as well as in levels of dopamine and its major metabolites, and those of serotonin metabolites in the hippocampus and striatum. In maternally deprived Long Evans rats treated with the probiotic mixture, the number of entries into the central area in the open-field test was increased, reflecting an anxiolytic effect. The probiotic mixture increased Tjp1 and decreased Ifnγ mRNA levels in the ileum of maternally deprived rats. In both models, probiotic supplementation changed the proportions of several Operational Taxonomic Units (OTU) in the gut microbiota, and the levels of certain cecal and serum metabolites were correlated with behavioral changes. Chronic administration of the tested probiotic mixture can therefore beneficially affect anxiety- and depressive-like behaviors in rats, possibly owing to changes in the levels of certain metabolites, such as 21-deoxycortisol, and changes in brain monoamines.

## Introduction

In addition to its well-known role in the development and maintenance of digestive, metabolic, and immune functions of the host, the gut microbiota is involved in the development and functioning of the central nervous system. Bidirectional communication between the gut microbiota and the central nervous system influences the development of the hypothalamic-pituitary-adrenal axis and stress reactivity. This has been well documented by studies in germ-free animals involving antibiotic or probiotic administration (Sudo et al., 2004; Crumeyrolle-Arias et al., 2014; see review in Luczynski et al., 2016). The gut microbiota also influences anxiety-and depression-like behaviors in rodents with differing effects according to their genetic constitution and their sensitivity to acute stress. When placed in a stressful environment, germ-free Swiss, NMRI and Kunming mice, which are moderately sensitive to stress, decreased anxiety-like behavior whereas germ-free Fischer 344 rats and BALB/C mice, which are more sensitive to stress, manifest an increase in anxiety-like behavior compared to their conventional counterparts (Diaz-Heijtz et al., 2011; Neufeld et al., 2011; Nishino et al., 2013; Crumeyrolle-Arias et al., 2014). Emotional responses following antibiotic-induced disturbances in the gut microbiota depend on the type of antibiotic. A mixture of antibiotics induced anxiolytic-like effects in conventional BALB/C mice subjected to light-dark box and the step-down tests (see review in Luczynski et al., 2016). Ampicillin increased anxiety-like behavior in the elevated plus maze and helplessness behavior in the forced swimming test in conventional BALB/C mice, whereas cefoperazone, belonging to a different antibiotic family, had no effect (Ceylani et al., 2018). Furthermore, modulatory effects of probiotics have been reported in several studies. Anxiolytic-like effects in BALB/C mice subjected to various behavioral tests were observed following treatment with several *Lactobacillus* and *Bifidobacterium* probiotic strains (Bercik et al., 2011; Bravo et al., 2011; Savignac et al., 2014). Similar findings were reported in the case of Wistar rats with regard to the conditioned defensive burying test (Messaoudi et al., 2011). In addition, *Lactobacillus rhamnosus* and *Lactobacillus helveticus* strains reversed the effects of maternal separation on neural circuits underpinning fear expression and extinction in infant Sprague Dawley rats (Cowan et al., 2019). The purpose of our study was to analyze the behavioral and biochemical effects of chronic oral administration of a probiotic mixture (M) of *Lactobacillus helveticus* LA 102, *Bifidobacterium longum* LA 101, *Lactococcus lactis* LA 103, and *Streptococcus thermophilus* LA 104 (Lactibiane Référence^®^, PiLeJe Laboratoire, France) in two experimental models of anxiety and depression. Since gut microbiota seems to modulate emotional responses depending on how animals react to stress, we studied the effects of M in experimental models comprising rats known to be hypersensitive to stress. The first experimental model was the Fischer rat, as this rat strain shows higher stress reactivity and depressive- and anxiety-like behaviors compared to other rat strains such as Sprague-Dawley or Wistar rats (Sudakov et al., 2001; Wu and Wang, 2010). The second experimental model, concerning Long Evans rats, was a model of maternal deprivation (pups separated from their mother and their littermates). In this strain, early environmental stress, such as maternal deprivation, leads to an increase in stress reactivity and anxiety-like behavior, as shown in the open-field test, as well as to opiate dependence in adulthood (Vazquez et al., 2005a,b). In Sprague-Dawley and Wistar rats, maternal deprivation separation in place of deprivation (pups being separated from their mother but not from their littermates) also led to dysbiosis, an increase in gut permeability, colon inflammation and induced visceral hyperalgesia (Barreau et al., 2004; O’Mahony et al., 2011; Pusceddu et al., 2015; Moya-Perez et al., 2017; Murakami et al., 2017).

Despite the abundance of data related to the ability of the gut microbiota to communicate with the central nervous system, and the effect of probiotics, the pathways and brain structures involved remain poorly understood. For this reason, in the present study, in addition to analyze emotional behavior in two experimental models after chronic treatment with M, we also quantified monoamine levels in the prefrontal cortex, striatum and hippocampus to assess possible correlations between behavioral responses and monoamine levels in these brain structures specifically involved in emotional behaviors. Furthermore, the impact of M on the levels of tight junction protein and inflammatory marker mRNAs was measured in the colon and ileum as an index of gut permeability. Finally, the composition of the microbiota in the feces, and the presence of certain metabolites in the cecal content and serum, were analyzed. The results of this study showed that chronic oral administration of M reduced anxiety-like and depressive-like behaviors, and modified gut microbiota and metabolites, in both Fischer rats and maternally-deprived Long Evans rats.

## Materials and Methods

### Animals

Fischer 344 male rats (*n* = 23), aged 4 weeks, were purchased from Charles River (Saint-Germain-Nuelles, France). Eleven Long Evans female rats on day 14 of gestation were obtained from Janvier (Le Genest-Saint-Isle, France) and male F1 Long Evans male rats were retained for the study (*n* = 48). Rats were housed by pairs, except for pregnant rats, which were housed individually, in a room maintained at 20–24°C with a 12-h light/dark cycle. All rats received food (RO3; Scientific Animal Food and Engineering, Augy, France) and water *ad libitum*.

Experimental procedures were carried out in accordance with EU Directive 2010/63/EU for animal experiments and approved by the Ethics Committee of the INRAE Research Center at Jouy-en-Josas and by the French Research Ministry (Approval no. 1239).

### Probiotic Mixture

M is composed of *Lactobacillus helveticus* LA 102, *Bifidobacterium longum* LA 101, *Lactococcus lactis* LA 103, and *Streptococcus thermophilus* LA 104 (Lactibiane Référence^®^, PiLeJe Laboratoire, Paris, France). It was dissolved extemporaneously in maltodextrin solution prior to administration. Both M and the excipient were provided by Genibio (Lorp-Sentaraille, France).

### Study Design

From 6 weeks of age onwards, rats received 0.5 mL of the excipient (control rats) or M (1.10^9^ CFU) by gavage with probes (Phymep, France) 5 days a week for 5 weeks for Fischer rats and 9 weeks for Long Evans rats (until euthanasia). Behavioral tests were conducted 15 days after the first gavage. They lasted 15 days for Fischer rats and 38 days for Long Evans rats. Fischer and Long Evans rats were tested during their light phase and first subjected to the novel object test, then to the black and white box, elevated plus maze, open-field and forced swimming tests (progressing from a less to more stressful environment). One or two behavioral tests were performed per week between 9:00 a.m. and 4:00 p.m. The tests and the gavage took twice as long for Long Evans rats since they were twice as many (48 rats). One week after the last test, rats were sacrificed and blood, brain, feces, cecal content and intestinal tissue were collected.

### Maternal Deprivation in Long Evans Rats

Maternal deprivation was performed as described by Vazquez et al. (2005b). The day of birth was designated as day 0. On postnatal day 1, litters were cross-fostered and culled to six to seven male pups. The pups were randomly attributed to foster dams to redistribute possible effects of genetic and prenatal factors and to obtain similar litter sizes. The litters were each assigned to an experimental group. From day 1, mothers were removed from their home cage and put in a new cage for 3 h/day, the same procedure being applied at each deprivation. Neonates belonging to the maternal deprivation group (D; *n* = 24) were individually placed in temperature- (30–34°C) and humidity-controlled cages divided into compartments. Pups’ cages contained 2 cm of fresh shavings covered with absorbent paper. Pups were isolated daily from day 1 to 14 from 1:00 to 4:00 p.m. They then return to their respective home cage and their mother was placed back in the cage. Pups not subjected to maternal deprivation (ND; *n* = 24) remained with their mother during this period and were handled just to change the bedding in their cages once a week. On day 22, all pups were weaned from their mothers and housed in pairs from the same litter.

### Behavioral Assessments

All behavioral tests were videotaped, and the data were analyzed by two experimenters blind to treatment.

#### Novel Object Test

This test was performed in a dimly lit dark gray open field (90 × 70 × 60 cm) containing an object (15 cm height) fixed (Patafix^®^) on the floor of the box. The latency time to the first visit to the object, the time spent close to the object and the number of visits to the object were recorded for 10 min (Ennaceur et al., 2005).

#### Light-Dark Box Test

The plastic test box (80 × 55 × 32 cm) was divided into two compartments of the same size, one black and topped with a black cover, the other white and illuminated (130 lux). The compartments were connected by a 10 × 10 cm opening located in the center of the partition. The rat was placed in the white area. The time spent in the white area, the number of transitions and the number of attempts to exit the dark compartment (head extended out of the dark side) were recorded for 10 min (Andrade and Graeff, 2001).

#### Elevated Plus Maze Test

The elevated plus maze was a dark gray apparatus comprising two open arms (50 × 10 cm), and two closed arms (50 × 10 × 50 cm). The maze was elevated to a height of 70 cm. The rat was placed in the center of the maze facing an open arm (Daugé et al., 1989). The number of visits into the open and closed arms and the time spent in these, the number of visits to the end of open arms and the number of head dippings and stretchings were recorded during 5 min.

#### Open Field Test

The same open field used in the novel object test was strongly illuminated (500 lux). Black lines on the floor delineated 10 ×10 cm squares (Daugé et al., 1988). The rats were placed in the same corner and videotracked for 6 min. The latency time to move from the initial corner was measured, as well as the number of rearings, squares crossed, defecations, groomings and entries into the central part of the field, considered as the most aversive section of the apparatus.

#### Forced Swimming Test

Rats were placed in a cylinder (diameter: 20 cm, height: 46 cm) filled with water (24 ± 1°C) up to 30 cm from the bottom (Naudon and Jay, 2005). The procedure consisted of a 15 min pre-test session followed by a 5 min test phase 24 h later, during which the immobility time was measured.

### Euthanasia, Fluid, and Tissue Collection

Rats were euthanized by decapitation. Brains were frozen in isopentane at −30°C. The blood was centrifuged (2,500 g, 20 min, 4°C), and the serum was frozen at −80°C. Ileal mucosa scrapings and colonic sections were collected in RNA later. The cecal content was weighed and the feces collected. All samples were stored at −80°C.

### Monoamines

Serial 150 μm thick coronal sections of the prefrontal cortex (PC), striatum and hippocampus were cut at −20°C using a cryostat microtome (Leica 3050S, Leica Microsystems, Germany). Sections were punched from the PC (anteroposterior, 4.6–2.6 mm from the bregma; lateral, 0–1 mm from bregma; dorsoventral, 3–6 mm from the skull), striatum (2.2–0.6 mm from the bregma; lateral, 1–4 mm from bregma; dorsoventral, 4–8 mm from the skull) and hippocampus (−2.5–4.6 mm from the bregma; lateral, 0–4 mm from bregma; dorsoventral, 2–4 mm from the skull) and the samples were frozen at −80°C (Paxinos and Watson, 1998).

The samples of the PC, striatum and hippocampus were homogenized by ultrasonication in 0.25 ml (PC, hippocampus) or 0.3 ml (striatum) of ice-cold 0.1 N perchloric acid containing 0.1% cysteine, using a Vibra Cell Sonicator (Sonics and Materials, Newtown, CT, United States), then centrifuged at 4°C for 10 min (18,000 g). The supernatants were filtered under pressure through 0.45 μm filters (Millipore, Ireland) and then kept frozen at −80°C until analysis. The pellets were resuspended in NaOH (0.1 M) and used for protein measurement (using the Bradford method).

The levels of dopamine (DA) and its metabolites 3,4-dihydroxyphenylacetic acid (DOPAC) and homovanillic acid (HVA), 3-methoxytyramine (3-MT), norepinephrine (NE), and serotonin (5-HT) and its metabolite 5-hydroxyindoleacetic acid (5HIAA) were determined in supernatants using a reversed-phase ion pair HPLC system with electrochemical detection (Leroux et al., 2014). The HPLC system consisted of a pump (Spectrasystem P1000 XR, Thermo Fisher Scientific, France) connected to a C18 reversed phase column Supelcosil (3.0 × 150 mm, 3 μm, Sigma-Aldrich, Bellefonte, PA, United States) coupled to an electrochemical detector (Decade II, Antec, Leyden, Netherlands) with a glassy carbon electrode set at 0.75 V vs. an Ag/AgCl reference electrode. The mobile phase consisted of 50 mM KH_2_PO_4_, 125 mL/L methanol, 0.5 mM octan-1-sulfonic acid sodium salt and 0.15 mM Na_2_EDTA at pH 3.9. The mobile phase was filtered through 0.45 μm cellulose acetate filters (Millipore) and delivered at a flow rate of 0.5 mL/min. Samples (20 μL) were injected into the HPLC system by means of an automatic device (AS3000, Thermo Electron Corporation, San Jose, CA, United States). Chromatograms were recorded and integrated by PC integration Azur software (Datalys, Le Touvet, France).

### Tight Junction Protein and Inflammatory Marker RNAs

RNAs of colonic and ileal mucosa were extracted using a Quiagen kit (Qiagen, France) according to the manufacturer’s instructions. RNAs were quantified and assessed for quality using an Agilent TM Bioanalyzer (Agilent, France). Reverse transcription was accomplished using an Applied Biosystems High Capacity cDNA Reverse Transcription kit (Thermo Fisher Scientific, France). Pre-amplification was performed using an Applied Biosystems TaqMan PreAmp Master Mix kit (Thermo Fisher Scientific, France) and predesigned TaqMan primers (Applied Biosystems, France) (Supplementary Table S1). The pre-amplified targeted genes were quantified using Q-PCR based on TaqMan gene expression assays with the predesigned TaqMan primers.

Q-PCR was performed on a Q-PCR machine StepOne Plus Applied Biosystems (Thermo Fisher Scientific, France). β-Actin was the housekeeping gene. Gene expression values were calculated using the comparative threshold cycle (Ct) method to generate ΔCt values. The relative abundance of each RNA was normalized according to the equation: Relative Quantity RQ = 2^–ΔΔ*Ct*^.

### Feces Microbiota

A modified version of the Godon et al. protocol (Godon et al., 1997) was used for feces DNA extraction. For each animal, 200 mg of the frozen fecal sample were resuspended in a mixture of 250 μL of guanidine thiocyanate buffer (4 M guanidine thiocyanate–0.1 M Tris (pH 7.5), 40 μL of 10% *N*-lauroyl sarcosine–0.1 M phosphate buffer (pH 8.0) and 500 μL of 5% *N*-lauroyl sarcosine; the tubes were then incubated at 70°C for 1 h. After addition of one volume (750 μL) of 0.1 mm diameter silica beads (Sigma), the tubes were shaken for 10 min at maximum speed on a Vibrobroyeur MM200 (Retsch, Germany). The tubes were then vortexed and centrifuged at 18,000 *g* for 5 min at 4°C. After recovery of the supernatant, 30 μL of Proteinase K (Chemagic STAR DNA BTS kit, Perkin Elmer, United States) were added and the samples were incubated for 10 min at 70°C at 250 rpm in a Multi-Therm (Benchmark Scientific, United States), then for 5 min at 95°C for enzyme inactivation. The tubes were then centrifuged at 18,000 *g* for 5 min at 4°C and the supernatants were transferred into the wells of a Deepwell plate. The plate was then placed on a Chemagic STAR nucleic acid workstation (Hamilton, Perkin Elmer, United States) and the DNA was extracted from the samples using a Chemagic STAR DNA BTS kit (Perkin Elmer, United States) according to the manufacturer’s instructions.

The V3-V4 regions of the 16S rDNA gene were amplified from the DNA extracts during the first PCR step using the fusion primers Vaiomer 1F and Vaiomer 1R (Nadkarni et al., 2002; Supplementary Table S2). The PCR (Lluch et al., 2015) was performed using 2 U of a DNA-free Taq DNA Polymerase and 1xTaq DNA polymerase buffer (MTP Taq DNA Polymerase, Sigma-Aldrich, United States). The buffer was completed with 10 nmol of dNTP mixture (Sigma-Aldrich, United States), 15 nmol of each primer (Eurofins) and Nuclease-free water (Qiagen, Germany) in a final volume of 50 μL.

The PCR reaction was carried out in a T100 Thermal cycler (Biorad, United States) as follows: an initial denaturation step (94°C for 10 min) was followed by 35 cycles of amplification (94°C for 1 min, 68°C for 1 min and 72°C for 1 min) and a final elongation step at 72°C for 10 min. Amplicons were then purified using a magnetic bead CleanPCR kit (Clean NA, GC biotech B.V., Netherlands) in a 96-well format. The concentration of the purified amplicons was checked using a Nanodrop spectrophotometer (Thermo Scientific, United States) and a subset of amplicon sizes was analyzed on a Fragment Analyzer (AATI, United States) with the reagent kit ADNdb 910 (35–1,500 bp). Sample multiplexing was performed by adding tailor-made 6 bp unique indexes during the second PCR step at the same time as the second part of the P5/P7 adapters to obtain the primer Vaiomer 2F and the reverse primer Vaiomer 2R (Supplementary Table S2). The second PCR (Lluch et al., 2015) step was performed on 50–200 ng of purified amplicons from the first PCR using 2.5 U of a DNA-free Taq DNA polymerase and 1xTaq DNA polymerase buffer. The buffer was completed with 10 nmol of dNTP mixture (Sigma-Aldrich, United States), 25 nmol of each primer (Eurofins, Luxembourg) and Nuclease-free water (Qiagen, Germany) up to a final volume of 50 μL. The PCR reaction was carried out on a T100 Thermal cycler with an initial denaturation step (94°C for 10 min), 12 cycles of amplification (94°C for 1 min, 65°C for 1 min and 72°C for 1 min) and a final elongation step at 72°C for 10 min. Amplicons were purified as described for the first PCR reaction. The concentration of the purified amplicons was measured using NanoDrop spectrophotometer (Thermo Fisher Scientific, United States) and the quality of a subset of amplicons (12 samples per sequencing run) was assessed on a Fragment Analyzer (AATI, United States) with the reagent kit ADNdb 910 (35–1,500 bp).

Checks were carried out to ensure that the large number of PCR cycles (35 cycles for PCR1 + 12 cycles for PCR2) had not generated significant amounts of PCR chimera or other artifacts. The region of the 16S rDNA gene to be sequenced had a length of 467 bp for a total amplicon length of 522 bp after PCR1 and of 588 bp after PCR2 (using the 16S rDNA gene of *E. coli* as a reference).

Negative controls to assess the technical background were included using Nuclease-free water (Qiagen, Germany) in place of the extracted DNA during library preparation.

All libraries were pooled to equal amounts in order to generate an equivalent number of raw reads for each library. The DNA concentration of the pool (no dilution, diluted 10x and 25x in EB + Tween 0.5% buffer) was quantified on a Qubit Fluorometer (Thermo Fisher Scientific, United States). The pool, at a final concentration between 5 and 20 nM, was used for sequencing.

The pool was denatured (NaOH 0.1N) and diluted to 7 pM. The PhiX Control v3 (Illumina, United States) was added to the pool at 15% of the final concentration as described in the Illumina procedure. Aliquots (600 μL) of this pool and the PhiX mixture were loaded onto the Illumina MiSeq cartridge according to the manufacturer’s instructions, using a MiSeq Reagent Kit v3 (2 × 300 bp Paired-End Reads, 15 Gb output). FastQ files were generated at the end of the run (MiSeq Reporter software, Illumina, United States) for quality control. The quality of the run was checked internally using PhiX Control and then each paired-end sequence was assigned to its sample using the multiplexing index.

Metabarcoding of the V3-V4 region of the 16S was performed using the Illumina MiSeq sequencing technology with the universal primers F343 (CTTTCCCTACACGACGCTCT TCCGATCTACGGRAGGCAGCAG) and R784 (GGAGTTCA GACGTGTGCTCTTCCGATCTTACCAGGGTATCTAATCCT). A total of 7,074,093 pairs from 118 samples (21,065–194,968 reads per sample) were provided by the sequencing platform. Quality controls of raw data were performed using FastQC v0.11.3 (Andrews, 2010) and no problems were detected. The pairs were then merged using Flash v1.2.11 (Magoc and Salzberg, 2011), the adapters were removed using cutadapt v1.12 (Martin, 2011) and the resulting sequences were cleaned using sickle v1.33 with the following filters (length > 20, no Ns, trimming of bases with quality lower than 20). In total, 84% of the pairs passed the initial quality control filters. The remaining sequences were then dereplicated and processed using the FROGS pipeline (Escudié et al., 2017) with default parameters. OTUs were clustered using Swarm v2.1.12 (Mahé et al., 2015) with parameter d = 3 and chimeras were filtered using vsearch v1.4 (Rognes et al., 2016) in *de-novo* mode. Finally, OTUs with low abundance (<50) and/or prevalence (found in less than eight samples) were filtered out, resulting in 2,576 OTUs (corresponding to 3,310,024 sequences). OTUs were affiliated by blasting cluster seed sequences against the Silva database v128 (Quast et al., 2013), 100% of the OTUs being affiliated, with at least 80% of identity and 80% of coverage between query and target.

### Short-Chain Fatty Acids (SCFA)

Cecal content samples were extracted in water and proteins were precipitated by adding phosphotungstic acid. The supernatant fraction (0.1 μL) was analyzed on a gas–liquid chromatograph (Autosystem XL; Perkin Elmer, Saint-Quentin-en-Yvelines, France) using 2-ethylbutyrate as the internal standard. Data were collected and peaks integrated using Turbochrom v. 6 software (Perkin Elmer, Courtaboeuf, France) (Lan et al., 2007).

### Liquid Chromatography Mass Spectrometry (LCMS) Metabolomics

#### Cecal Content Samples

Samples of cecal content (25 mg) were homogenized in 150 mL cooled methanol (at −20°C) for 1 min using a ball mill (Retsh^®^, France) at a frequency of 50 Hz. The homogenized samples were vortexed, incubated at −20°C for 30 min, and then centrifuged for 15 min (11,000 *g*, 4°C). The supernatant was filtered through 10 kDa filter tubes by centrifuging for 45 min (11,000 g, 4°C). The extracts obtained were dried in a stream of nitrogen and frozen at −80°C. All the dried polar extracts were reconstituted with 150 μL acetonitrile/water (50:50 v:v) and analyzed using a UPLC ultimate 3000 system (Thermo Scientific), coupled to a high-resolution Q-Exactive Plus mass spectrometer (Martin et al., 2015). Metabolites were identified by reference to an in-house database, including details of more than 800 metabolites with their chromatographic retention time.

#### Serum Samples

Serum samples were processed essentially as described by Rosique and colleagues (Rosique et al., 2019). LCMS analysis was performed as described above.

### Statistical Analyses

The data obtained in all experiments (except those concerning the microbiome and metabolomics) were analyzed using ANOVA and Student’s *t*-test for pairwise comparisons in the case of a normal distribution, and the Kruskal-Wallis and Mann and Whitney tests for pairwise comparisons in the case of a non-normal distribution (GraphPad Prism software, version 5.04, La Jolla, CA, United States). An emotionality *Z*-score corresponding to behavioral modifications (head dipping and exit attempts for Fischer rats, visits to the central area of the open field and rearings for Long Evans rats) was calculated (Guilloux et al., 2011).

Data concerning the feces microbiota were analyzed using R software (R Core Team, 2017), specifically Rstudio and the following R packages: ggplot2 v1.0 (Wickham, 2016) and phyloseq (McMurdie and Holmes, 2013). The raw (OTU) table comprised a total of 2,576 OTUs from 118 samples (data not shown). Before computing alpha and beta-diversity indices, samples were rarefied to the same read depth of 11,098, corresponding to the sample with the fewest reads. Local alpha-diversity was estimated through the richness and Inverse-Simpson indices. The effect of rat strain, M supplementation, and maternal deprivation on α-diversity was then tested by ANOVA. Beta-diversity was estimated by the Bray-Curtis index and Permanova (using the adonis function from the VEGAN package) (Dixon, 2003) was used to test the effect of rat strain, M supplementation, and maternal deprivation on beta-diversities. To limit the confounding effect of rat strains, and at the cost of statistical power, we split the samples by rat strain before performing the differential abundance analysis. We also filtered OTU based on prevalence (>50% in one combination of maternal deprivation x probiotic administration) and relative abundance (>0.1% in at least one sample) resulting in 407–437 OTUs per rat strain. Differential abundance tests were performed using DESeq2 (Love et al., 2014) and OTUs with adjusted *p* < 0.1 were declared differentially abundant. For Long Evans rats, we used DESeq2 to test (i) the effect of maternal deprivation using only control samples (i.e., from rats not supplemented with M), (ii) the effect of M and M x maternal deprivation interaction using all samples. For Fischer rats, as there was no maternal deprivation, we estimated only the effect of M supplementation.

Metabolomic data were analyzed using PLS regression methods and univariate statistics using SIMCAP12 (Sartorius, Aubagne, France) and metaboanalyst (Xia et al., 2009).

The level of significance was set at *p* < 0.05.

## Results

### Fischer Rats

#### Behavioral Assessments

##### Novel Object Test

There were no significant differences between M-supplemented and control rats in latency to the first visit to the novel object (*U* = 59.5, *p* = 0.7), number of visits (*U* = 58.0, *p* = 0.6) or time spent visiting the object (*U* = 66.0, *p* = 0.9).

##### Light-Dark Box Test

**FIGURE 1:**
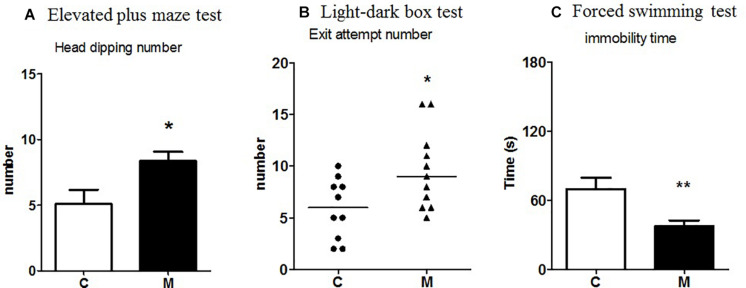
Effect of the probiotic mixture in Fischer rats on **(A)** number of head dippings in the elevated plus maze test; **(B)** number of exit attempts in the light-dark box test; **(C)** immobility time (s) in the forced swimming test. C, control group (*n* = 10–12); M, group receiving the probiotic mixture (*n* = 11–12). The results are expressed as the mean ± SEM for the number of head dippings and immobility time and as the median and interquartile range for the number of exit attempts. *p < 0.05, **p < 0.01 vs. C.

There was an increase in the number of exit attempts from the black compartment to the white one made by M-supplemented rats compared to control rats (*U* = 25.5, *p* = 0.04) (Figure 1B). No significant effect was observed with regard to time spent in the white compartment (*U* = 45.5, *p* = 0.3) or number of transitions (*U* = 42.5, *p* = 0.5).

##### Elevated Plus Maze Test

Compared to control rats, M-supplemented rats showed significantly more head dippings in the open arms (*t* = 2.37, *p* = 0.02; Figure 1A), whereas no significant effect was observed in the percentage of visits (*t* = 0.03, *p* = 0.9), the time spent in the open arms (*t* = 0.51, *p* = 0.9), the number of stretchings (*t* = 0.61, *p* = 0.5), or the number of visits to the end of the open arms (*t* = 0.29, *p* = 0.7).

##### Open Field Test

There were no significant differences between M-supplemented and control rats in the number of squares crossed (*U* = 57.5, *p* = 0.8), entries into the central area (*U* = 59.5, *p* = 0.9), rearings (*U* = 56.5, *p* = 0.8), groomings (*U* = 52.5, *p* = 0.6), or defecations (*U* = 58.0, *p* = 0.9), or in the latency time (*U* = 53.0, *p* = 0.6).

##### Forced Swimming Test

M-supplemented rats showed a significant decrease in immobility time compared to control rats (t = 3.0, p = 0.005) (Figure 1C).

### Brain Monoamines

There were no significant differences between M-supplemented and control rats as regards monoamine levels in the PC (not shown). M supplementation significantly decreased DA (*U* = 24.0, *p* = 0.05), DOPAC (*U* = 14.0, *p* = 0.002) and HVA levels (*U* = 7.0, *p* = 0.0005) in the hippocampus as well as DA (*U* = 27.0, *p* = 0.03) and DOPAC (*U* = 11.5, *p* = 0.001) levels and ratio 5HIAA/5HT (*U* = 20, *p* = 0.009) in the striatum (Table 1A). In addition, there was a negative correlation between the increase in the number of head dippings in the elevated plus maze and DA (Spearman *r* = −0.81, *p* = 0.003) and DOPAC (Spearman *r* = −0.76, *p* = 0.008) levels in the hippocampus (not shown).

**TABLE 1:** Effects of the probiotic mixture on monoamine, metabolite (ng/mg proteins) and turnover (%) levels in the hippocampus and striatum of **(A)** Fischer, **(B)** maternally deprived Long Evans rats.

**(A)**				

**Hippocampus**		**Control**		**Probiotic mixture**
Dopamine		2.7 (3.2)		0.83 (1.4)*
DOPAC		2.3 (2.5)		0.75 (0.8)**
HVA		6.2 (3.9)		3.3 (0.8)***
DOPAC/DA		98.7 (61.7)		83.7 (64.3)
HVA/DA		241.2 (151.8)		318.9 (199.6)
5HT		2.1 (1.1)		1.8 (0.7)
5HIAA		6.5 (3.4)		5.4 (2.3)
5HIAA/5HT		300.5 (108.1)		302.4 (57.9)
NE		3.1 (2.1)		3.0 (1.8)
**Striatum**		
Dopamine		145.6 (59.5)		107.2 (51.7)*
3-MT		2.8 (1.1)		2.6 (2.1)
DOPAC		13.4 (4.8)		8.9 (3.1)***
HVA		19.9 (5.4)		15.9 (9.6)
DOPAC/DA		9.3 (2.9)		8.5 (2.7)
HVA/DA		13.1 (4.4)		14.4 (5.9)
3-MT/DA		1.7 (0.4)		2.1 (1.1)
5HT		0.9 (1.6)		0.8 (1.1)
5HIAA		0.9 (1.1)		0.3 (0.7)
5HIAA/5HT		71.5 (46.5)		39.3 (28.5)**
NE		8.1 (6.5)		9.0 (6.7)

**(B)**				

	**NDC**	**NDM**	**DC**	**DM**

**Hippocampus**				
DA	4.4 (6.6)	4.5 (4.6)	1.0 (0.8)***	2.4 (2.9)**^++^
DOPAC	1.7 (3.0)	2.1 (3.7)	0.5 (0.6)	0.8 (1.0)^++^
HVA	6.8 (5.1)	6.2 (8.4)	2.3 (2.3)**	4.0 (4.4)^+^
DOPAC/DA	40.0 (24.7)	40.8 (29.9)	56.3 (49.1)	63.9 (55.9)
HVA/DA	134.4 (53.8)	150.4 (86.7)	233.5 (344.9)	211.0 (192.5)
5HT	2.6 (3.2)	2.7 (2.4)	1.2 (1.5)**	1.0 (1.0)**^++^
5HIAA	7.2 (7.1)	6.4 (5.4)	4.9 (4.1)*	4.0 (3.9)**^+^
5HIAA/5HT	277.0 (155.5)	275.4 (180.5)	403.2 (161.6)**	474.9 (227.3)**^++^
NE	7.4 (6.3)	7.0 (8.1)	3.4 (2.6)***	4.0 (2.1)**^++^
**Striatum**				
DA	128.7 (55.1)	161.3 (72.8)	166.6 (72.5)*	194.1(59.2)***^+^
3-MT	0.9 (0.6)	1.4 (0.8)	1.7 (0.7)***	1.7 (0.4)***^++^
DOPAC	13.5 (8.9)	15.3 (10.6)	19.0 (11.0)**	28.7 (9.8)**^++#^
HVA	6.2 (4.1)	6.8 (4.3)	12.7 (8.6)**	10.9 (6.2)**^++^
DOPAC/DA	11.4 (4.2)	11.7 (3.1)	11.7 (1.9)	14.7 (5.0)
HVA/DA	4.8 (2.3)	4.9 (1.6)	6.4 (3.9)	5.9 (3.9)
3-MT/DA	0.8 (0.4)	0.9 (0.3)	1.0 (0.2)	0.9 (0.2)
5HT	0.3 (0.3)	0.4 (0.4)	0.4 (0.4)	0.4 (0.3)
5HIAA	0.5 (0.3)	0.5 (0.3)	0.5 (0.4)	0.7 (0.4)
5HIAA/5HT	112.1 (117.0)	115.2 (62.8)	120.6 (70.8)	139.9 (64.9)
NE	7.0 (6.3)	4.9 (6.0)	3.6 (4.8)	3.4 (4.2)

Mann and Whitney test, n = 11–12, *p < 0.05, **p < 0.01, ***p < 0.001 vs. C. DA, dopamine; 3-MT, 3-methoxytyramine; DOPAC, 3,4-dihydroxyphenylacetic acid; HVA, homovanillic acid; 5HT, serotonine; 5HIAA, 5-hydroxyindolacetic acid; NE, norepinephrine. The results are expressed as median and interquartile range. ND, non deprived; D, deprived; C, control; M, probiotic mixture. Mann and Whitney test, n = 12, *p<0.05, **p < 0.01, ***p < 0.001 vs. NDC, ^+^p < 0.05, ^++^p < 0.01 vs. NDM, ^#^p < 0.05 vs. DC.

### Tight Junction Protein and Inflammatory Marker mRNAs in the Gut Mucosa

RNAs of tight junction protein 1 (Tjp1), occludin 1 (OCEL1) and claudin 2 (Cldn2) as well as those of the inflammatory markers interleukin 10 (Il-10) and interferon γ (Ifnγ) were detected in the ileum and colon. M supplementation induced a significant decrease in Tjp1 (*U* = 15, *p* = 0.001) and Cldn2 (*U* = 15, *p* = 0.001) mRNAs in the colon compared to the levels of these proteins in the colon of non-supplemented control rats, but no difference was seen with regard to the ileum (Table 2A).

**TABLE 2:** Effects of the probiotic mixture on junction protein (Tjp1, OCEL1, Cldn2) and inflammatory marker (TNF-α, Ifnγ, Il-10) RNAs in the ileum and colon of **(A)** Fischer and **(B)** maternally deprived Long Evans rats.

**(A) Fischer rats**						

**Ileum**						

	**Tjp1**	**OCEL1**	**Cldn2**	**TNF-α**	**Ifnγ**	**Il-10**
C	1.0 (0.5)	0.96 (0.3)	1.0 (0.3)	1.1 (0.9)	1.1 (0.6)	0.58 (1.2)
M	0.99 (0.4)	0.97 (0.4)	1.1 (0.7)	1.1 (0.7)	1.3 (1.2)	1.3 (0.9)

**Colon**						

	**Tjp1**	**OCEL1**	**Cldn2**	**TNF-α**	**Il-10**	

C	1.0 (0.3)	0.87 (0.6)	1.0 (0.5)	1.0 (0.7)	1.1 (0.9)	
M	0.67 (0.3)***	0.81 (0.4)	0.47 (0.3)***	0.77 (0.6)	0.97 (0.7)	

**(B) Long Evans rats**						

**Ileum**						

	**Tjp1**	**OCEL1**	**Cldn2**	**TNF-α**	**Ifnγ**	**Il-10**

NDC	0.99 (0.2)	0.93 (0.4)	0.99 (0.5)	1.02 (0.5)	0.86 (0.8)	1.06 (0.6)
NDM	0.85 (1.3)	1.14 (0.5)	1.19 (0.4)	0.86 (0.4)	1.59 (1.6)	0.79 (0.5)
DC	0.59 (0.2)***	1.11 (0.5)	1.03 (0.3)	1.12 (0.8)	2.90 (4.9)**	0.57 (0.7)
DM	0.88 (0.4)++	1.36 (0.5)	1.18 (0.5)	1.13 (0.8)	1.69 (4.8)	0.56 (0.7)

**Colon**						

	**Tjp1**	**OCEL1**	**Cldn2**	**TNF-α**	**Il-10**	

NDC	0.88 (0.4)	0.97 (0.3)	1.01 (0.6)	0.98 (1.1)	0.78 (0.7)	
NDM	1.18 (0.7)*	1.42 (0.7)**	1.06 (1.1)	0.64 (0.8)	0.92 (1.0)	
DC	0.94 (0.3)	1.33 (0.6)**	1.15 (0.7)	0.48 (0.3)	0.59 (0.4)	
DM	1.15 (0.7)+	1.17 (1.2)	1.33 (0.6)	1.25 (1.1)	1.14 (1.2)	

Results are expressed as 2^–ΔΔ*Ct*^. Mann and Whitney test. n = 11–12. ***p = 0.001 vs. C. Mann and Whitney test n = 11–12, *p < 0.05, **p< 0.01, **p < 0.01 vs. NDC, +p < 0.05, ++p < 0.01 vs. DC.

*ND, non deprived rats; D, deprived rats; C, control; M, probiotic mixture; Tjp1, tight junction protein 1; OCEL1, occludin 1; Cldn2, claudin 2; TNF-α, tumor necrosis factor; Ifnγ, interferon γ; Il-10, interleukine 10. The results are expressed as median and interquartile of 2^–ΔΔ*Ct*^ divided by the median of control group.*

### Fecal Microbiota

**FIGURE 2:**
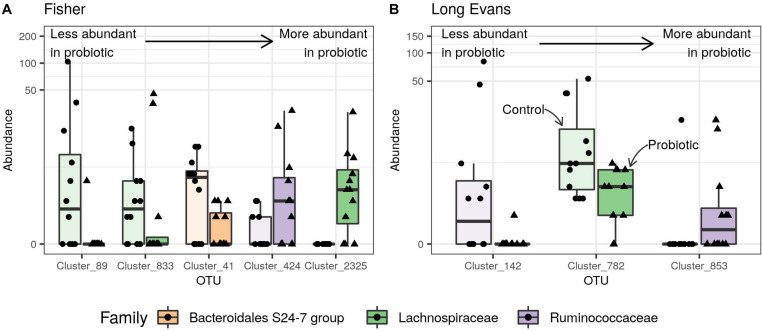
Effect of the probiotic mixture on the normalized abundance of Differentially Abundant (DA) fecal microbial OTUs in **(A)** Fischer rats, **(B)** maternally deprived Long Evans rats. Points represent abundances in individual samples and boxplots indicate the median value and the interquartile range. Transparent boxplots correspond to control samples and opaque boxplots to samples from rats supplemented with the probiotic mixture. DA-OTUs are colored according to their rank and sorted by size effect: from less to more abundant in samples from probiotic-supplemented rats. Clusters 89, 833, and 2,325 were defined as *Lachnospiraceae* NK4A136 genus **(A)** and cluster 853 as *Butyricoccus* genus **(B)**.

There was no significant variation in the α- and β-diversities between the two groups of rats [ANOVA of observed richness, *F*_(1,22)_ = 3.68, *p* = 0.06; Permanova, *F*_(1,22)_ = 1.14, *p* = 0.2, respectively]. Nevertheless, differential abundance analysis revealed five OTUs belonging to the *Bacteroidales*, *Lachnospiraceae*, and *Ruminococcaceae* that differed between the two groups: two OTUs from the genus *Lachnospiraceae* NK4A136 and one from an unknown genus were decreased in M-supplemented rats compared to the control group whereas one OTU from the genus *Lachnospiraceae* NK4A136 and one OTU of an unknown genus were increased in the M-supplemented group compared to the control group (Figure 2A).

### Cecal Short-Chain Fatty Acids

M-supplementation induced a significant decrease in acetate as a percentage of total SCFA levels in the cecal content (*U* = 32, *p* = 0.03). Other SCFA and total SCFA levels did not differ between the groups (Supplementary Table S3A).

### Metabolomics

A partial Least-square discriminant analysis (PLS-DA) was performed on the 121 annotated cecal metabolites to determine the effect of M. Statistical validations are provided in Supplementary Material. Applying a variable selection with a variable importance in projection (VIP) factor cut-off of 1.12, a robust discriminant model was calculated with 39 cecal metabolites (*p* = 0.00056 after cross-validation ANOVA) (Supplementary Figure S1A). At the individual scale, no metabolite appeared to be modified by M supplementation. Of the 39 metabolites identified, a combination of 25 was found to be associated with the *Z*-score in a PLS regression (*p* = 0.01) (Supplementary Figure S2A). Among these, only three were found to be associated with the *Z-*score at the individual level (Supplementary Table S4A), methylthioadenosine, thymidine and 2-acetamido-2-deoxy-beta-D-glucosylamine (Supplementary Figure S2B). No combination appeared to be related to the forced swimming behavior.

### Long Evans Rats

#### Behavioral Assessments

##### Novel object test

ND and D rats did not differ significantly with regard to latency to the first visit to the novel object (*H* = 4.34, *p* = 0.22) or the number of visits (*H* = 8.7, *p* = 0.03; pairwise comparisons were not significant). In contrast, the time spent visiting the object was significantly decreased in D compared to ND rats (*U* = 17.5, *p* = 0.01). M supplementation did not change behavioral parameters.

##### Light-Dark Box Test

The time spent in the white compartment and the numbers of transitions and exit attempts did not differ significantly between the four groups (time spent: *H* = 3.62, *p* = 0.3; transitions: *H* = 3.80, *p* = 0.2; exit attempts: *H* = 1.9, *p* = 0.3).

##### Elevated Plus Maze Test

Maternal deprivation and M supplementation did not significantly affect the percentage of visits to the open arms or the percentage of time spent in these open arms [% of visits: deprivation factor: *F*_(1,44_ = 0, treatment factor: *F*_(1,44)_ = 0.19, *p* = 0.6, interaction: *F*_(1,44)_ = 0.01, *p* = 0.9]; % of time spent: deprivation factor: *F*_(1,44)_ = 0.01, *p* = 0.9, treatment factor: *F*_(1,44)_ = 0.13, *p* = 0.7, interaction: *F*_(1,44)_ = 0.14, *p* = 0.7), or the numbers of visits to the end of the open arms [deprivation factor: *F*_(1,44)_ = 0.5, *p* = 0.4, treatment factor: *F*_(1,44)_ = 0.02, *p* = 0.88, interaction: *F*_(1,44)_ = 1.08, *p* = 0.3], head dippings [deprivation factor: *F*_(1,44)_ = 0.18, *p* = 0.6, treatment factor: *F*_(1,44)_ = 0.83, *p* = 0.3, interaction: *F*_(1,44)_ = 0.3, *p* = 0.5] and stretchings [deprivation factor: *F*_(1,44)_ = 0.16, *p* = 0.6, treatment factor: *F*_(1,44)_ = 0.32, *p* = 0.5, interaction: *F*_(1,44)_ = 0.3, *p* = 0.5].

##### Open field test

**FIGURE 3:**
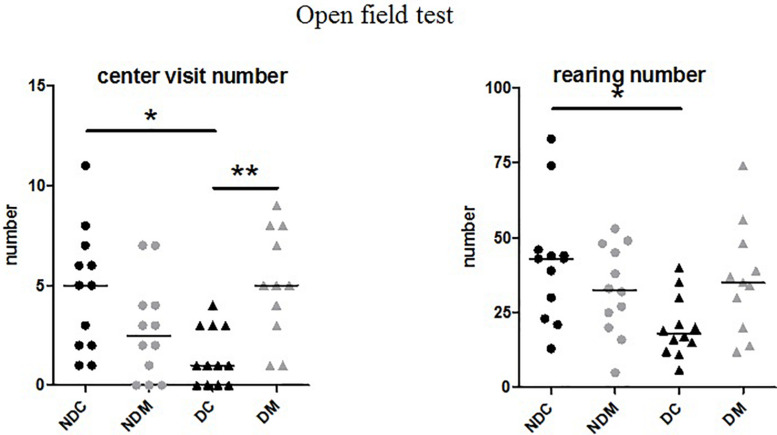
Effect of the probiotic mixture on the number of visits to the central area and the number of rearings in the open field test in maternally deprived Long Evans rats. NDC, not maternally deprived control group (*n* = 12); NDM, not maternally deprived group receiving the probiotic mixture (*n* = 12); DC, maternally deprived control group (*n* = 12); DM, maternally deprived group receiving the probiotic mixture (*n* = 12). The results are expressed as the median and interquartile range. Number of visits to the central area (*H* = 13.7, *p* = 0.003), number of rearings (*H* = 10.2, *p* = 0.01) *p < 0.05 vs. NDC, **p < 0.01 vs. DC.

The number of rearings and visits to the central area were significantly different between the four groups (rearings: *H* = 10.2, *p* = 0.01; central area: *H* = 13.7, *p* = 0.003). Maternal deprivation decreased the number of rearings (*U* = 19, *p* = 0.01) and visits to the central area (*U* = 22.5; *p* = 0.01). In D rats, M increased the numbers of rearings (*U* = 31.0, *p* = 0.03) and visits to the central area (*U* = 15.0, *p* < 0.001; Figure 3). The latency time (*H* = 3.14, *p* = 0.37) and the numbers of squares crossed (*H* = 6.48, *p* = 0.09), groomings (*H* = 4.58, *p* = 0.20) and defecations (*H* = 1.42, *p* = 0.70) did not differ between the four groups (ND control, NDM, D control, DM).

##### Forced Swimming Test

There were no statistically significant differences between the four groups with respect to immobility time [deprivation: *F*_(1, 44)_ = 0.95, *p* = 0.3, treatment: *F*_(1,44)_ = 0.13, *p* = 0.7, interaction: *F*_(1,44)_ = 0.04, *p* = 0.8].

### Brain Monoamines

Monoamine levels in the PC did not differ significantly between the four groups (not shown). In the hippocampus, there were significant differences between the four groups in the levels of DA (*H* = 18.5, *p* = 0.0004), DOPAC (*H* = 9.3, *p* = 0.02), HVA (*H* = 13.9, *p* = 0.003), 5HT (*H* = 29.5, *p* = 0.0001) and NE (*H* = 15.3, *p* = 0.001) and in the 5HIAA/5HT ratio (*H* = 15.3, *p* = 0.001). Maternal deprivation decreased the contents of DA (*U* = 12, *p* = 0.001), HVA (*U* = 20, *p* = 0.01), 5HT (*U* = 28, *p* = 0.01), and NE (*U* = 16, *p* = 0.001) and increased the 5HIAA/5HT ratio (*U* = 22, *p* = 0.01). Long Evans rats receiving M did not show significant differences compared to their control NDC and DC counterparts. In the striatum, the levels of 5HT and NE and monoamine turnover were not significantly modified but there were significant differences between the four groups in the levels of DA (*H* = 12.6, *p* = 0.005), 3-MT (*H* = 17.3, *p* = 0.0006), DOPAC (*H* = 19.1, *p* = 0.0003), and HVA (*H* = 16.5, *p* = 0.0009). Maternal deprivation induced an increase in DA (*U* = 35, *p* = 0.03), 3-MT (*U* = 13.5, *p* = 0.0008), DOPAC (*U* = 31, *p* = 0.01), and HVA (*U* = 20, *p* = 0.006) levels. M supplementation did not significantly modify the levels measured in the NDC and DC groups (Table 1B).

### Tight Junction Protein and Inflammatory Marker RNAs

The mRNAs of Tjp1, OCEL1, Cldn2, Il-10, and Ifnγ were detected in the ileum and colon. In the colon, OCEL1 mRNAs and Tjp1 mRNAs levels were significantly modified between the four groups (OCLE1: *H* = 7.88, *p* = 0.04; Tjp1: *H* = 8.59, *p* = 0.03). In the colon, maternal deprivation induced a significant increase in OCEL1 mRNAs (*U* = 31, *p* = 0.01), M inducing a significant increase in Tjp1 (*U* = 37, *p* = 0.04) in this group. M also induced a significant increase in Tjp1 (*U* = 34, *p* = 0.03) and OCEL1 RNA levels (*U* = 30, *p* = 0.01) in the colon of ND rats. In the ileum, Tjp1 and Ifnγ RNA levels were significantly different between the four groups (Tjp1: *H* = 14.5, *p* = 0.002; Ifnγ: *H* = 9.1, *p* = 0.02). In the ileum, maternal deprivation significantly decreased Tjp1 expression levels (*U* = 13, *p* = 0.0007) and increased Ifnγ RNA levels (*U* = 12, *p* = 0.009), whereas M induced the opposite effects (Tjp1:*U* = 30, *p* = 0.01). M had no effects on RNA levels in the ileum of ND rats (Table 2B). In addition, there was a positive correlation between the time spent visiting a novel object and Tjp1 expression in the ileum of DM rats (Spearman r = 0.51, *p* = 0.04).

### Fecal Microbiota

Analysis of β-diversity revealed significant differences between Fischer and Long Evans rats with respect to the overall composition of the fecal microbiota [Permanova, species: *F*_(1,68)_ = 42,42, *p* = 0.0001; Supplementary Figure S3A].

In Long Evans rats alone, there was no significant variation in α-diversity between the four groups [ANOVA of observed richness, D: *F*_(1,44)_ = 3.94, *p* = 0.053, M: *F*_(1,44)_ = 0.05, *p* = 0.81, interaction: *F*_(1,44)_ = 0.29, *p* = 0.58]. β-diversity analysis demonstrated that maternal deprivation had a significant impact on the overall composition of the fecal microbiota [Permanova, D: *F*_(1,44)_ = 2.974, *p* = 0.0006, M: *F*_(1,44)_ = 1.37, *p* = 0.11, interaction: *F*_(1,44)_ = 0.84, *p* = 0.64, Supplementary Figure S3B].

Differential abundance analysis indicated that maternal deprivation modified the abundance of 47 genera belonging to the Bacteroidetes, Firmicutes and Proteobacteria phyla compared to the ND control group. The D control group showed a decrease in *Lachnospiraceae* NK4A136 (1 OTU), as well as in *Oscillibacter* (1 OTU), *Parabacteroides* (1 OTU), *Roseburia* (2 OTUs), *Ruminiclostridium* (1 OTU), *Ruminiclostridium* 5 (1 OTU), *Ruminiclostridium* 9 (1 OTU), *Ruminococcaceae* UGC-014 (1 OTU) and unknown genera (5 OTUs). The D control group showed an increase in *Anaerotruncus* (1 OTU), *Bacteroides* (3 OTUs), *Coprococcus* 1 (1 OTU), *Desulfovibrio* (1 OTU), *Lachnoclostridium* (2 OTUs*), Lachnospiraceae* NK4A136 (11 OTUs), multi-affiliation (2 OTUs), *Oscillibacter* (1 OTU), *Prevotellaceae* UCG-001 (2 OTUs), *Ruminococcaceae* UGC-014 (2 OTUs) and unknown genera (7 OTUs) (Supplementary Figure S4). M supplementation did not significantly modify the abundance of any bacteria in the ND group but did so for 3 OTUs from the *Lachnospiraceae and Ruminococcaceae* family in the D group, inducing an increase in *Butyricicoccus* (1 OTU) and *Oscillibacter* (1 OTU) and a decrease in unknown genera (2 OTUs) (Figure 2B).

### Cecal Short-Chain Fatty Acids

#### Maternal Deprivation Had No Significant Effect on SCFA Levels

The percentage of caproate was significantly different between the four groups (*H* = 13.6, *p* = 0.003). M induced a significant decrease in the level of caproate in the cecal content as a percentage of total SCFA level compared to the DC group (*U* = 16, *p* = 0.002). Levels of other SCFA and total SCFA levels did not differ between the four groups (Supplementary Table S3B).

#### Metabolomics

**FIGURE 4:**
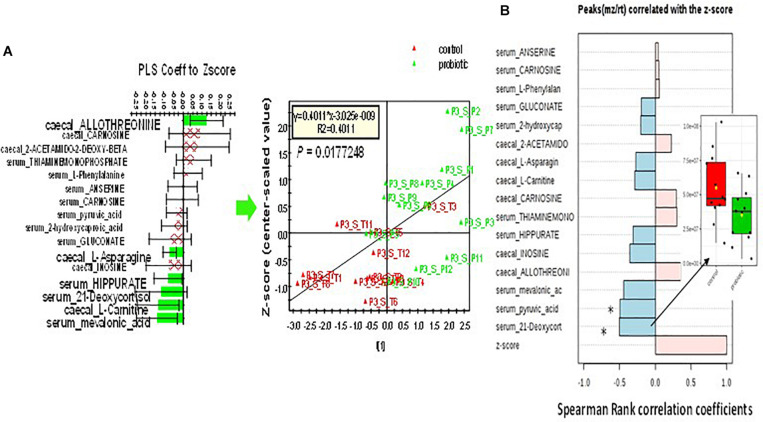
Correlation of a selection of 16 cecal and serum metabolites in Long Evans DM rats with *Z*-score. **(A)** PLS regression of a combination of 6 out of the 16 selected metabolites to *Z*-score (*p* = 0.01 after cross-validation using ANOVA). **(B)** Individual Pearson correlation with *Z*-score. Serum 21-deoxycortisone and pyruvic acid both displayed a significant individual relationship to behavior and sensitivity to M (all adjusted values of *q* < 0.1). Insert: relative quantity of serum 21-deoxycortisol in control (red box) and treated (green box) rats.

A PLS-DA was performed on 121 and 131 annotated cecal and serum metabolites to detect any effect of M. Statistical validations are provided in Supplementary Material. A similar analysis was performed to select metabolites associated with maternal deprivation (Supplementary Figures S1B,C). A total of 16 metabolites in the cecal content and serum that were associated with both maternal deprivation and sensitivity to M were retained and analyzed for correlation with the Z-score (Supplementary Table S4B). A combination of 6 out of the 16 metabolites was found to be significantly associated with the Z-score in a PLS regression analysis. Serum 21-deoxycortisol and pyruvic acid were found to be both collectively and individually related to both maternal deprivation and M (Figure 4).

## Discussion

This study showed that chronic oral administration of M induced behavioral effects and modified gut microbiota and metabolites both in naturally stress-sensitive Fischer rats and in maternally-deprived Long Evans rats.

In Fischer rats, M did not modify the global motor activity in the elevated plus maze and in open-field test but increased the number of exit attempts in the light-dark box and head dippings in the open arms of the elevated plus maze. These findings could mean that M induced a slight decrease in anxiety-like behavior, since the classical parameters (time spent in the light compartment of the light-dark box and percentage of the number of visits and of time spent in the open arms of the elevated plus maze) were unchanged. The increase of head dippings may also suggest risk-taking behavior. An association of *Lactobacillus helveticus* (R0052) and *Bifidobacterium longum* (R0175) was also reported to decrease anxiety-like behavior in Wistar rats in the conditioned defensive burying test (Messaoudi et al., 2011). In contrast to the subtle effects of M on anxiety-like behavior, M decreased immobility time in the forced swimming test, indicating potential antidepressant-like properties.

M decreased dopaminergic transmission in the hippocampus and both dopaminergic and serotoninergic transmission in the striatum. Fischer rats are known to be hyper-reactive to stress and to present hyperactivity of the dopaminergic systems (Sziraki et al., 2001; Wu and Wang, 2010). A decrease in DA synthesis and/or the number of DA neurons induced by M could indicate a decrease in anxiety-like behavior for Fischer rats. In addition, there was a negative correlation between the increase in the number of head dippings in the elevated plus maze and DA and DOPAC levels in the hippocampus. This is in agreement with the role of the hippocampus in modulating fear and anxiety-like behaviors. This brain structure is connected to the septum, locus coeruleus, raphe nuclei, hypothalamus, amygdala and medial frontal cortex, all these cerebral regions being involved in anxiety. Furthermore, the hippocampus also receives dopaminergic afferences from the ventral tegmental area and may be substantia nigra, and both dorsal and ventral parts of the hippocampus are implicated in anxiety-like behavior through the activation of D1 and/or D2 receptors (Bast and Feldon, 2003; Nasehi et al., 2011; review in Zarrindast and Khakpai, 2015; Feng et al., 2018). Conversely, a lesion of the hippocampus or local administration of DA antagonists in the hippocampus induced a decrease in anxiety-like behavior (Zarrindast and Khakpai, 2015).

M also decreased Tjp1 and Cldn2 RNA levels in the colon. Usually, the greater the amount of tight junction proteins present, the more intestinal permeability is reduced. This seems to be the case for Tjp1 (Van Itallie and Anderson, 2014), but the opposite was found for Cldn2 (Zeissig et al., 2007). It is therefore difficult to draw any conclusion regarding this relationship without measuring both Tjp1 and Cldn2 protein levels and membrane permeability. None of the bacterial species constituting M was found in the rat feces but M treatment modified the abundance of five OTUs belonging to the Bacteroidetes and Firmicutes phyla. An increase or a decrease was observed in *Lachnospiraceae* NK4A136 abundance, as well as in that of unknown genera. The bacterial SCFA acetate level was decreased in the cecal content of M rats without any correlation with behaviors. On the other hand, the metabolites methylthioadenosine, thymidine and 2-acetamido-2-deoxy-beta-D-glucosylamine appeared to be individually correlated with the Z-score. The first two metabolites can be synthesized by *Lachnospiraceae* but as yet, no data are available concerning their behavioral effects.

Finally, M supplementation of Fischer rats induced anxiolytic- and antidepressant-like effects, which could be due in part to a decrease in dopaminergic transmission in the hippocampus and to the presence of certain host and bacterial metabolites in the intestinal content. M also acted on intestinal physiology and modified the abundance of certain bacterial strains in the gut microbiota.

In Long Evans rats, maternal deprivation, as expected, induced a decrease in the number of entries into the central part of the open-field (Vazquez et al., 2005a), and also in rearing behavior. In the novel object test, the time spent visiting the object was also reduced. We could not exclude that this could be due to a reduced movement in the arena. However, we did not observe changes in the number of squares crossed and rearing in the open-field test. The behavioral changes observed in the open-field test were completely suppressed by M, indicating anxiolytic-like properties. Although some data from the literature reported an anxiogenic-like effect in the elevated plus maze test, after maternal separation (Wang et al., 2020), we have never found such effect in our experimental conditions (deprivation model, 3h of deprivation from day 1 to 14). Lacidofil^®^ and *Bifidobacterium pseudocatenulatum* CECT 7765 administered during the maternal separation period were reported to suppress, respectively, fear and anxiety-like behaviors in infant rodents (Cowan et al., 2016; Moya-Perez et al., 2017). Taken together, these results show that some probiotics, including M have preventive properties with regard to anxiety-like behavior but also may act on constitutive anxiety-like behavior.

As expected, maternal deprivation did not modify monoamine levels in the PC (Lejeune et al., 2013). However, it led to a decrease in catecholaminergic transmission and/or neurons and increased serotonergic neuronal activity in the hippocampus, as indicated with the increased 5HT turnover, whose ratio 5HIAA/5HT is an index. The elevated 5HT turnover could be an adaptation to compensate the decrease in other monoaminergic transmissions and/or neuronal loss. Dopaminergic transmission was also increased in the striatum. Although few data have been published regarding monoamine levels in the brain of maternally separated/deprived Long Evans rats, Matthews et al. (2001) found higher DA levels in the striatum and lower 5HT levels in the hippocampus of Lister-Hooded rats maternally separated for 6 h/day.

However, no correlation was found between behavioral changes and monoamine levels in either brain structure. M did not modify levels of monoamines and their metabolites in any of the three structures in either ND or D rats.

The higher OCEL1 mRNA levels in the colon of D rats could indicate an unexpected decrease in gut permeability but its role at tight junctions is still controversial. Notably, OCEL knockout mice have normal gut permeability and apparently normal tight junction. This protein might play an indirect role in permeability regulation (Van Itallie and Anderson, 2014). Maternal deprivation seemed to induce, as expected, a deterioration of the intestinal barrier with a decrease in Tjp1 RNA levels and an increase in expression of the pro-inflammatory marker Ifnγ in the ileum. Previous data concerning Sprague-Dawley rats showed that maternal separation increases the permeability of the intestinal barrier and also induces low-grade inflammation and greater microbial translocation, leading to functional deterioration of the intestine (Gareau et al., 2008; O’Mahony et al., 2011; Ganguly and Brenhouse, 2015). In the present study, chronic administration of M suppressed the abnormalities observed in the colon and ileum of D rats, indicating a potential protective effect on the intestinal barrier. In addition, there was a positive correlation between the time spent visiting a novel object and Tjp1 expression in the ileum of DM rats.

Maternal deprivation modified the abundance of 47 genera belonging to the Bacteroidetes, Firmicutes and Proteobacteria phyla. The maternal separation model has also been used as a model of irritable bowel syndrome, but the results obtained regarding gut microbiota composition are conflicting (Lejeune et al., 2013; Pusceddu et al., 2015; Murakami et al., 2017; Fukui et al., 2018). It is therefore conceivable that it is not the bacterial species that are important but rather their metabolic activities. In our study, none of the bacteria constituting M was found in the feces of rats but in D rats, M modified the abundance of certain bacterial species with an increase in members of the *Butyricicoccus* and *Oscillibacter* genera, and a decrease in bacteria belonging to unknown genera. *Butyricicoccus pullicaecorum* has been shown to be a potential probiotic in patients with inflammatory bowel disease and to attenuate colitis in rats (Eeckhaut et al., 2013; Steppe et al., 2014). *Oscillibacter* is known to produce anti-inflammatory metabolites (Lino et al., 2007; Man et al., 2011; Arpaia et al., 2013). There was also a decrease in caproate level in the cecal content of DM rats without any correlation with behaviors. Levels of certain metabolites in the cecal content and serum of maternally deprived rats differed according to whether the rats had been supplemented with M and these differences were correlated with behavior. Among them serum 21-deoxycortisol, a barely described metabolite in rat was reported to occur through adrenal mitochondrial CYP21A1 from 17α-hydroxyprogesterone (Maschler and Horn, 1976). 21-deoxycortisol has been shown to behave as a glucocorticoid receptor agonist (Pijnenburg-Kleizen et al., 2015) and for the first time we have shown its increase after maternal deprivation. Interestingly, its decrease in rats supplemented with M was associated with a decrease in anxiety-like behavior in D rats although cortisol was not detected and serum corticosterone levels remained unchanged (data not shown).

Finally, in Long Evans rats, M induced anxiolytic-like effects that were correlated with changes in certain host and bacterial metabolites, seemed to enhance the intestinal barrier, and modified the profile of the gut microbiota.

It is interesting to note that although Fischer and maternally deprived Long Evans rats showed distinct anxiety-like behaviors (fear of emptiness and light in Fischer rats and fear of large spaces and/or light in Long Evans rats), M was effective in reducing these behaviors in all cases.

## Conclusion

In conclusion, the probiotic mixture tested (M) can beneficially affect anxiety- and depressive-like behaviors in both naturally stress-sensitive Fischer rats and maternally deprived Long Evans rats. M modified the composition of the gut microbiota, intestinal physiology in Fischer and Long Evans rats and brain monoamines in Fischer rats. intestinal tight junction protein expression and some cecal and serum levels of certain metabolites in Fischer and Long Evans rats were observed, reinforcing our knowledge of the links between the gut microbiota and the brain. The next step will be to investigate the behavioral effects of methylthioadenosine, thymidine, 2-acetamido-2-deoxy-beta-D-glucosylamine and 21-deoxycortisol in our experimental models and to determine their mechanisms of action.

## Data Availability Statement

The original contributions presented in the study are publicly available. This data can be found here: https://www.ncbi.nlm.nih.gov/sra/PRJNA668659 and https://www.ebi.ac.uk/metabolights/, ID: MTBLS2126.

## Ethics Statement

The animal study was reviewed and approved by the Ethics Committee of the INRAE Research Center at Jouy-en-Josas and by the French Research Ministry (Approval No. 1239).

## Author Contributions

CP, MMa, OR, M-NR, LS, FT, MMo, and DJ designed the work that led to the submission, acquired data, drafted the manuscript, and approved the final version. J-CM and ND designed the work, acquired data, played a role in interpreting the results, drafted the manuscript, and approved the final version. MB and SH played an important role in interpreting the results, revised the manuscript, and approved the final version. SR and LN conceived and designed the work, played an important role in interpreting the results, revised the manuscript, and approved the final version. VD conceived and designed the work, acquired data, played an important role in interpreting the results, drafted and revised the manuscript, and approved the final version. All authors contributed to the article and approved the submitted version.

## Conflict of Interest

SH and MB were employees of PiLeJe Laboratoire. The remaining authors declare that the research was conducted in the absence of any commercial or financial relationships that could be construed as a potential conflict of interest.
