# Supplementary material for: A Probiotic Mixture Induces Anxiolytic- and Antidepressive-Like Effects in Fischer and Maternally Deprived Long Evans Rats
Source: Front Behav Neurosci. 2020 Nov 12;14:581296. doi: 10.3389/fnbeh.2020.581296 (PMC7708897; doi:10.3389/fnbeh.2020.581296)
Supplement: Supplementary file 1 [file Data_Sheet_1.docx]

**Supplemental statistical information for selected figures.**

Complementary statistical data of PLS analyses, method described in Erickson, K. L., Trygg, J., & Wold, S. (2008). CV-ANOVA for significance testing of PLS and OPLS1 models. *J. Chemometrics, 22*, 594–600.

**SS**, sum of squares, **DF**, degree of freedom, **MS**, mean square (e.g. SS/DF), **SD**, standard deviation, **p**, probability

**Figure 4A.**

Cross validation ANOVA PLS details

|  | **SS** | **DF** | **MS** | **F** | **p** | **SD** |
| --- | --- | --- | --- | --- | --- | --- |
| z-score |  |  |  |  |  |  |
| **Total corr** | 22 | 23 | 0.956522 |  |  | 0.978019 |
| **Regression** | 7.01624 | 2 | 3.50812 | 4.91669 | 0.0177248 | 1.873 |
| **Residual** | 14.9838 | 21 | 0.713513 |  |  | 0.844697 |

Permutation test

Observations are randomly permuted to either group, 200 times. The resulting explained (R2Y, green) and predicted (Q2Y, blue) variances must decreased at the y intercept when false observation group assignments are provided along with the number of permutations (from right to left).

**Figure S1a**

Cross validation ANOVA PLS-DA statistical details

|  | **SS** | **DF** | **MS** | **F** | **p** | **SD** |
| --- | --- | --- | --- | --- | --- | --- |
| **Total corr.** | 22 | 22 | 1 |  |  | 1 |
| **Regression** | 8.88622 | 2 | 4.44311 | 6.77625 | 0.00566308 | 2.10787 |
| **Residual** | 13.1138 | 20 | 0.655689 |  |  | 0.809746 |

Permutation test

0.0

0.2

0.4

0.6

0.8

1.0

0.0

0.1

0.2

0.3

0.4

0.5

0.6

0.7

0.8

0.9

1.0

Intercepts: R2=(0.0, 0.434), Q2=(0.0, -0.123)

200 permutations 1 components

R2

Q2

**Figure S2a**

Cross validation ANOVA PLS details

| **M18(z-score select CS)** | **SS** | **DF** | **MS** | **F** | **p** | **SD** |
| --- | --- | --- | --- | --- | --- | --- |
| **Zscore** |  |  |  |  |  |  |
| **Total corr** | 20 | 20 | 1 |  |  | 1 |
| **Regression** | 7.84297 | 2 | 3.92149 | 5.80625 | 0.0113286 | 1.98027 |
| **Residual** | 12.157 | 18 | 0.67539 |  |  | 0.821821 |

Permutation test (200)

0.0

0.2

0.4

0.6

0.8

1.0

0.0

0.2

0.4

0.6

0.8

1.0

R2=(0.0, 0.375), Q2=(0.0, -0.13)

200 permutations 1 components

R2

Q2

**Figure S1bA**

Cross validation ANOVA PLS-DA statistical details

|  | **SS** | **DF** | **MS** | **F** | **p** | **SD** |
| --- | --- | --- | --- | --- | --- | --- |
| **Total corr.** | 118 | 118 | 1 |  |  | 1 |
| **Regression** | 42.3083 | 4 | 10.5771 | 15.9302 | 2.18598e-010 | 3.25224 |
| **Residual** | 75.6917 | 114 | 0.663962 |  |  | 0.814839 |

Permutation test (200)

**Figure S1bB**

Cross validation ANOVA PLS-DA statistical details

|  | **SS** | **DF** | **MS** | **F** | **p** | **SD** |
| --- | --- | --- | --- | --- | --- | --- |
| **Total corr.** | 47 | 47 | 1 |  |  | 1 |
| **Regression** | 14.9588 | 4 | 3.73969 | 5.01874 | 0.00207549 | 1.93383 |
| **Residual** | 32.0412 | 43 | 0.745145 |  |  | 0.863218 |

Permutation test (200 permutations)

**Figure S1cA**

Cross validation ANOVA PLS-DA statistical details

|  | **SS** | **DF** | **MS** | **F** | **p** | **SD** |
| --- | --- | --- | --- | --- | --- | --- |
| **Total corr.** | 23 | 23 | 1 |  |  | 1 |
| **Regression** | 11.9124 | 2 | 5.95619 | 11.281 | 0.000470609 | 2.44053 |
| **Residual** | 11.0876 | 21 | 0.527982 |  |  | 0.726624 |

Permutation test (200 permutations)

**Figure S1cB**

Cross validation ANOVA PLS-DA statistical details

|  | **SS** | **DF** | **MS** | **F** | **p** | **SD** |
| --- | --- | --- | --- | --- | --- | --- |
| **Total corr.** | 48 | 48 | 1 |  |  | 1 |
| **Regression** | 20.9573 | 4 | 5.23933 | 8.52469 | 3.49394e-005 | 2.28896 |
| **Residual** | 27.0427 | 44 | 0.614606 |  |  | 0.783968 |

Permutation test (n=200)
