# Supplementary material for: A Probiotic Mixture Induces Anxiolytic- and Antidepressive-Like Effects in Fischer and Maternally Deprived Long Evans Rats
Source: Front Behav Neurosci. 2020 Nov 12;14:581296. doi: 10.3389/fnbeh.2020.581296 (PMC7708897; doi:10.3389/fnbeh.2020.581296)
Supplement: Supplementary file 2 [file Table_1.docx]

**Table S1**: Taqman assay

|  |  |  |
| --- | --- | --- |
| **Name (*Gene*)** | **Gene Bank accession n°** | **Assay ID** |
| Actb | NM_031144 | Rn00667869-m |
| Gapgh | NM_017008 | Rn01775763-g1 |
| Il-10 | NM_012854 | Rn01483988-g1 |
| Tnfα | NM_012675 | Rn01525859-g1 |
| Ifnγ | NM_138880 | Rn00594078-m1 |
| Il-13 | NM_053828 | Rn00587615-m1 |
| Tjp1 (ZO-1) | NM_001106266 | Rn02116071-s1 |
| Cldn2 | NM_001106846 | Rn02063575-s1 |
| Ocel1 | NM_001106065 | Rn01420322-g1 |
| Mylk3 | NM_001110810 | Rn01538099-m1 |

Actb = actin beta, Gapgh = glyceraldehyde phosphate deshydrogenase, Il-10 = interleukin 10, Tnf-α = tumor necrosis factor α, Ifnγ = interferon γ, IL-13 = interleukin 13, Tjp1 = tight junction protein 1, Cldn2 = claudin 2, Ocel1 = occludin 1, Mylk3 = myosin light chain kinase 3.
