# Supplementary material for: A Probiotic Mixture Induces Anxiolytic- and Antidepressive-Like Effects in Fischer and Maternally Deprived Long Evans Rats
Source: Front Behav Neurosci. 2020 Nov 12;14:581296. doi: 10.3389/fnbeh.2020.581296 (PMC7708897; doi:10.3389/fnbeh.2020.581296)
Supplement: Supplementary file 3 [file Table_2.DOCX]

**Table S2:** Sequences of primers

| Primers | Sequence |
| --- | --- |
| Vaiomer 1F | CTTTCCCTACACGACGCTCTTCCGATCT-TCCTACGGGAGGCAGCAGT  partial P5 adapter–primer |
| Vaiomer 1R | GGAGTTCAGACGTGTGCTCTTCCGATCT-GGACTACCAGGGTATCTAATCCTGTT  partial P7 adapter–primer |
| Vaiomer 2F | AATGATACGGCGACCACCGAGATCTACACT-CTTTCCCTACACGAC  partial P5 adapter–primer targeting primer 1F |
| Vaiomer 2R | CAAGCAGAAGACGGCATACGAGAT-NNNNNN-GTGACT-GGAGTTCAGACGTGT  partial P7 adapter including index–primer targeting primer 1R |

Primers Vaiomer 1F and 1R are specific for the 16S rDNA gene of 95% of the bacteria in the Ribosomal Database Project and part of the P5/P7 adapter targeted by the second PCR step (CTTTCCCTACACGAC and GGAGTTCAGACGTGT).
