# Supplementary material for: A Probiotic Mixture Induces Anxiolytic- and Antidepressive-Like Effects in Fischer and Maternally Deprived Long Evans Rats
Source: Front Behav Neurosci. 2020 Nov 12;14:581296. doi: 10.3389/fnbeh.2020.581296 (PMC7708897; doi:10.3389/fnbeh.2020.581296)
Supplement: Supplementary file 4 [file Table_3.DOCX]

**Table S3** : Effects of the probiotic mixture on short chain fatty acid (SCFA) levels in the caecal content of **A**) Fischer and **B**) maternally deprived Long Evans rats.

**A)** Acetate Propionate Butyrate Valerate Caproate

C 54.0 (3.0) 7.8 (1.8) 34.4 (4.9) 0.94 (0.1) 1.41 (0.5)

M 52.1 (5.1)* 8.1 (1.7) 36.7 (5.1) 1.00 (0.1) 1.5 (0.1)

Isobutyrate Isovalerate Isocaproate total Iso total SCFA

C 0.55 (0.3) 0.39 (0.3) 0 0.94 (0.5) 82.9 (24.5)

M 0.61 (0.3) 0.51 (0.2) 0 1.10 (0.5) 89.1 (14.9)

Mann and Whitney test **p* <0.05 vs. C.

**B)** Acetate Propionate Butyrate Valerate Caproate

NDC 54.4 (8.5) 7.2 (3.3) 33.5 (8.7) 0.95 (0.2) 1.42 (0.2)

NDM 58.5 (4.9) 8.2 (1.5) 29.9 (7.4) 0.87 (0.2) 1.2 (0.6)

DC 56.8 (7.1) 7.8 (1.0) 31.9 (8.1) 0.92 (0.1) 1.5 (0.3)

DM 57.8 (8.2) 8.9 (2.8) 30.4 (11.0) 0.99 (0.3) 1.1 (0.8)**

Isobutyrate Isovalerate Isocaproate total Iso total SCFA

NDC 0.57 (0.2) 0.38 (0.2) 0 0.96 (0.3) 78.0 (17.2)

NDM 0.5 (0.1) 0.35 (0.1) 0 0.86 (0.2) 70.4 (20.8)

DC 0.42 (0.2) 0.26 (0.2) 0 0.68 (0.4) 78.3 (10.1)

DM 0.48 (0.1) 0.3 (0.1) 0 0.75 (0.2) 77.1 (18.1)

Results are expressed as median and interquartile of percentage of short chain fatty acid over total SCFA (%) except total SCFA expressed in µmol/g. ND = non deprived, D = deprived, C = control, M = probiotic mixture. Kruskal Wallis test (caproate: *H* = 11.27, *p*=0.01) ***p*<0.01 vs. DC.
