# Supplementary material for: A Probiotic Mixture Induces Anxiolytic- and Antidepressive-Like Effects in Fischer and Maternally Deprived Long Evans Rats
Source: Front Behav Neurosci. 2020 Nov 12;14:581296. doi: 10.3389/fnbeh.2020.581296 (PMC7708897; doi:10.3389/fnbeh.2020.581296)
Supplement: Supplementary file 5 [file Table_4.DOCX]

Table S4: Values of Zscore (data normalized for head dippings in the elevated plus maze test + exit attempts in the light-dark box test)

A) FISCHER

M

2 1,35

3 0.53

4 0.66

5 0.5

6 0.72

7 0.08

8 0.66

9 0.91

10 1.77

12 1.70

Controls

1 0.05

2 -1.57

3 0.37

4 -0.01

5 0.095

6 0.10

7 0.11

8 0.54

9 0.06

10 0.34

11 0.08

Values of Zscore (data normalized for the number of visits in the center of the open-field + number of rearings in the open-field).

B) Long-Evans

Maternal deprivation

1 -1.29

2 -1.34

3 -0.17

4 -1.34

5 -0.58

6 -1.66

7 -1.24

8 -1.37

9 -1.27

10 -1.30

11 -0.46

12 -0.90

Maternal deprivation + M

1 0.39

2 1.30

3 -0.42

4 0.18

5

6 -0.16

7 1.02

8 0.18

9 -0.04

10 -1.34

11 -0.97

12 -1.15
