# Supplementary material for: A Probiotic Mixture Induces Anxiolytic- and Antidepressive-Like Effects in Fischer and Maternally Deprived Long Evans Rats
Source: Front Behav Neurosci. 2020 Nov 12;14:581296. doi: 10.3389/fnbeh.2020.581296 (PMC7708897; doi:10.3389/fnbeh.2020.581296)

## Slide 1
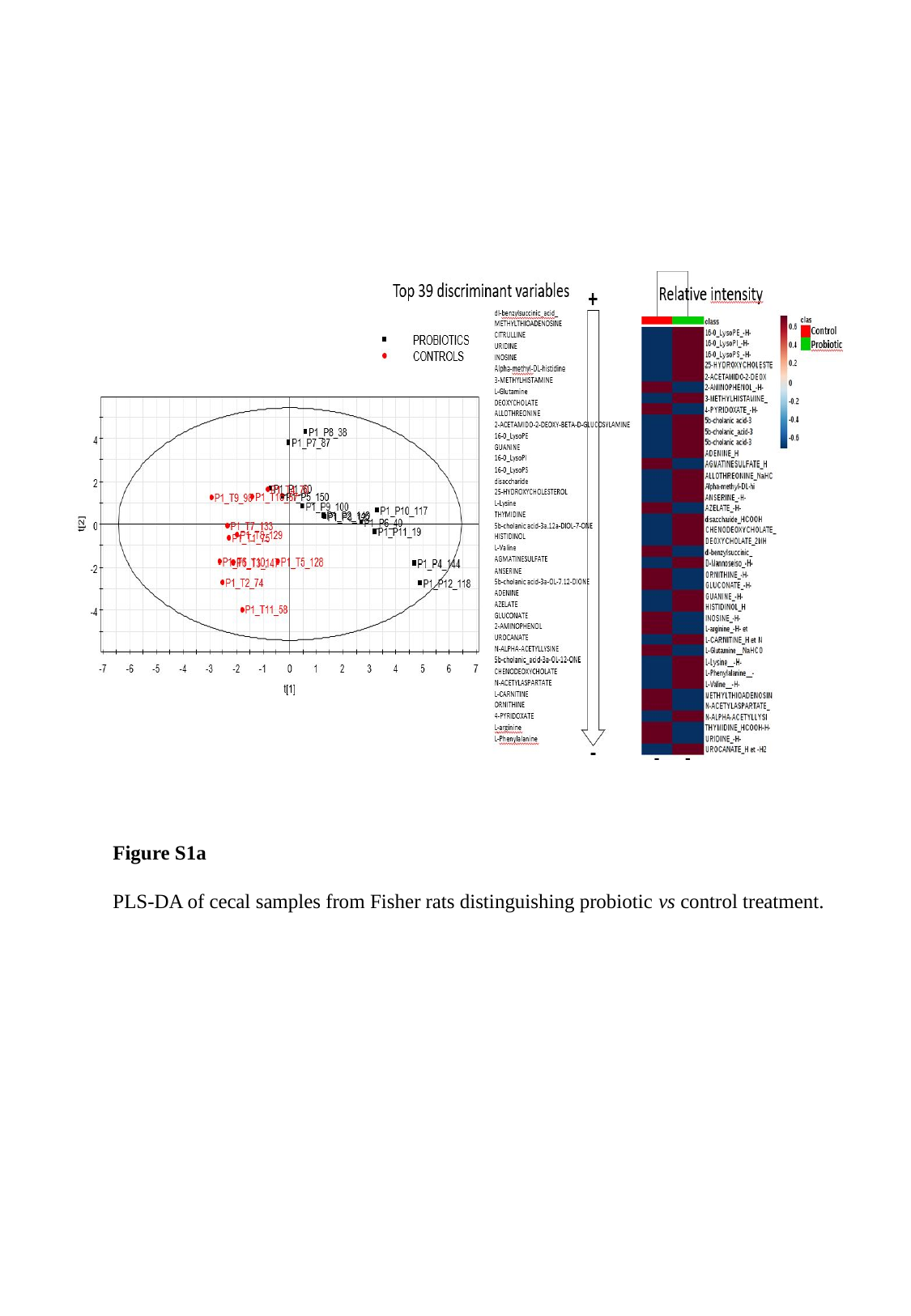

Figure S1a
PLS-DA of cecal samples from Fisher rats distinguishing probiotic vs control treatment.

## Slide 2
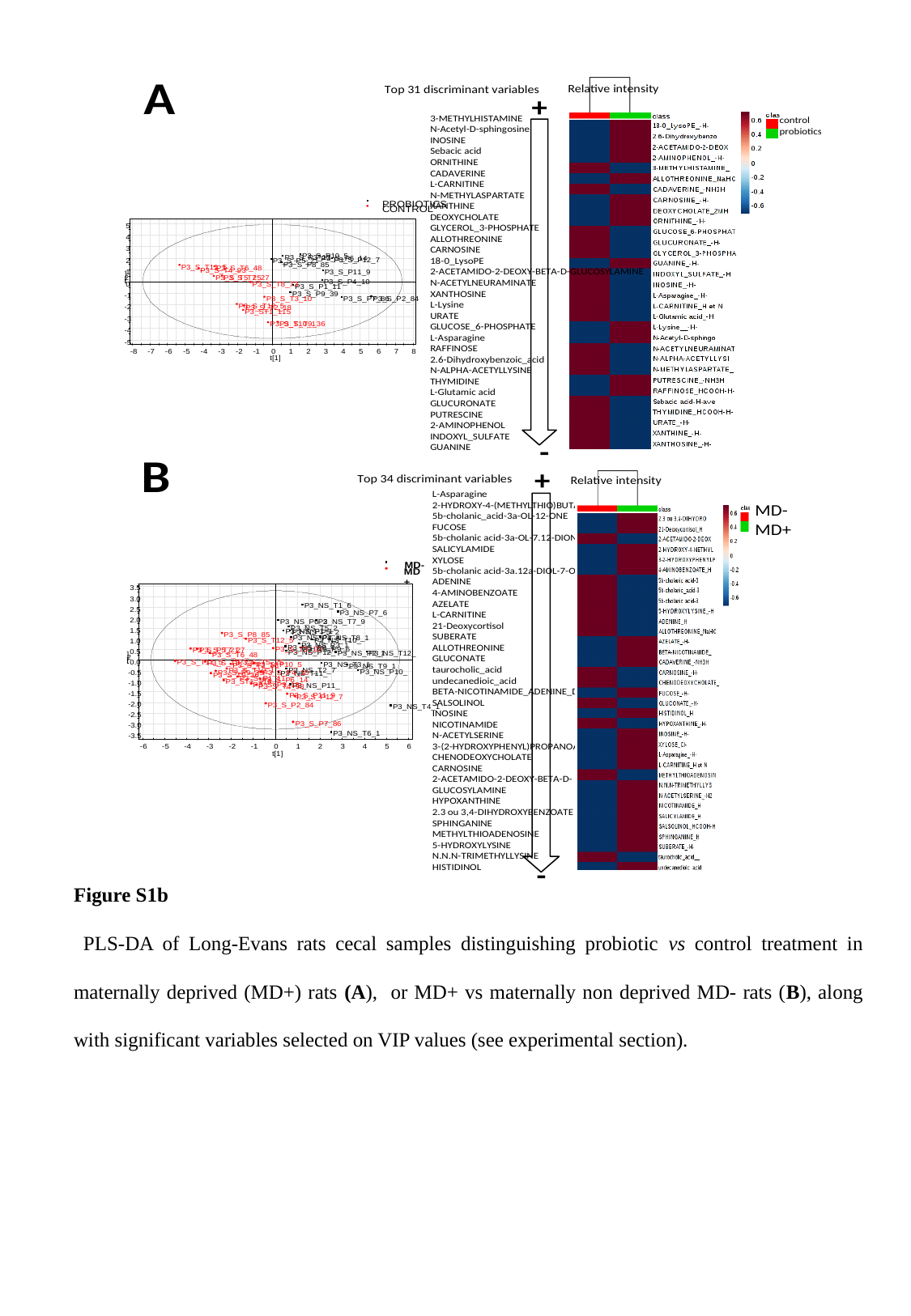

Figure S1b
 PLS-DA of Long-Evans rats cecal samples distinguishing probiotic vs control treatment in maternally deprived (MD+) rats (A), or MD+ vs maternally non deprived MD- rats (B), along with significant variables selected on VIP values (see experimental section).

## Slide 3
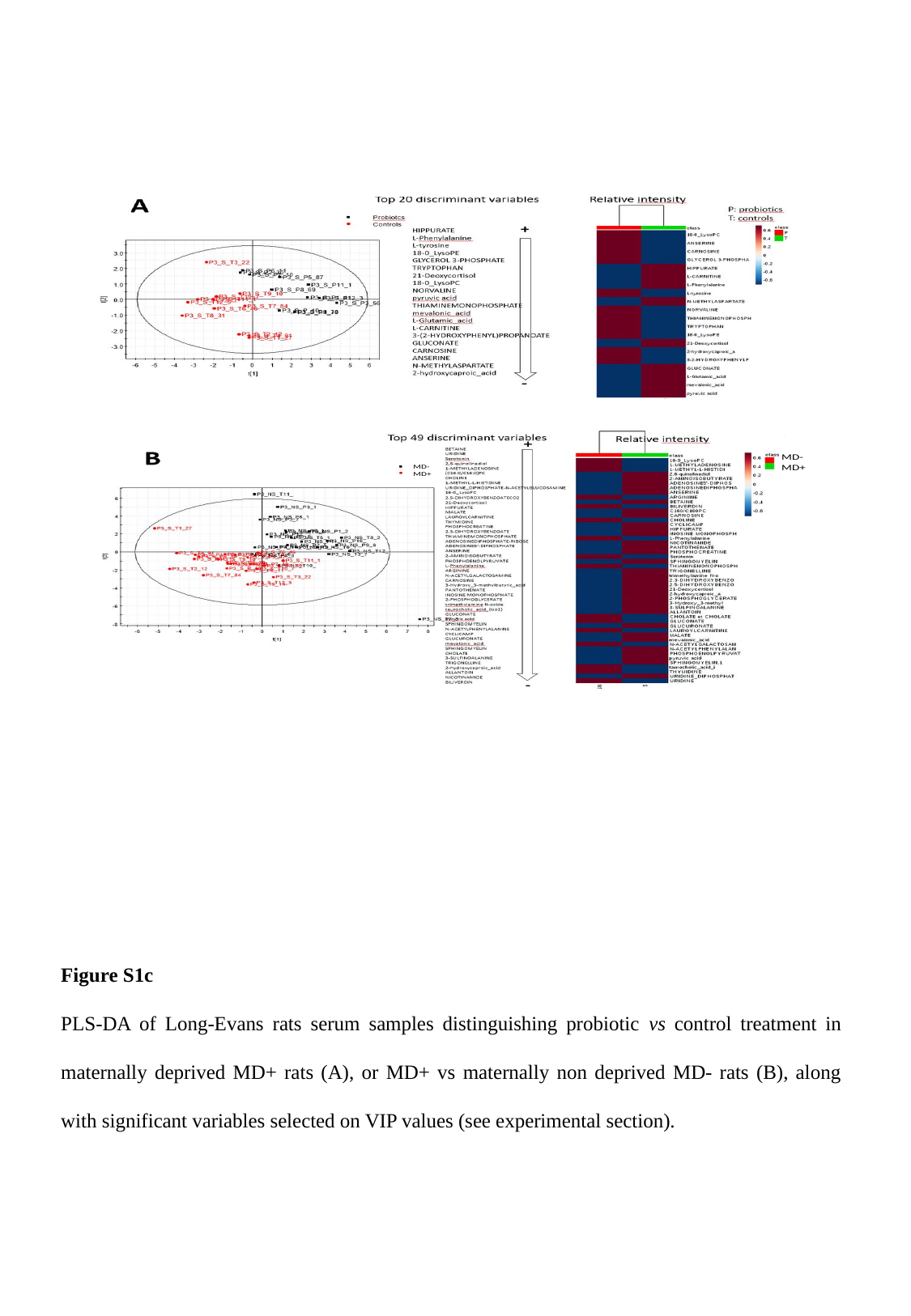

Figure S1c
PLS-DA of Long-Evans rats serum samples distinguishing probiotic vs control treatment in maternally deprived MD+ rats (A), or MD+ vs maternally non deprived MD- rats (B), along with significant variables selected on VIP values (see experimental section).

## Slide 4
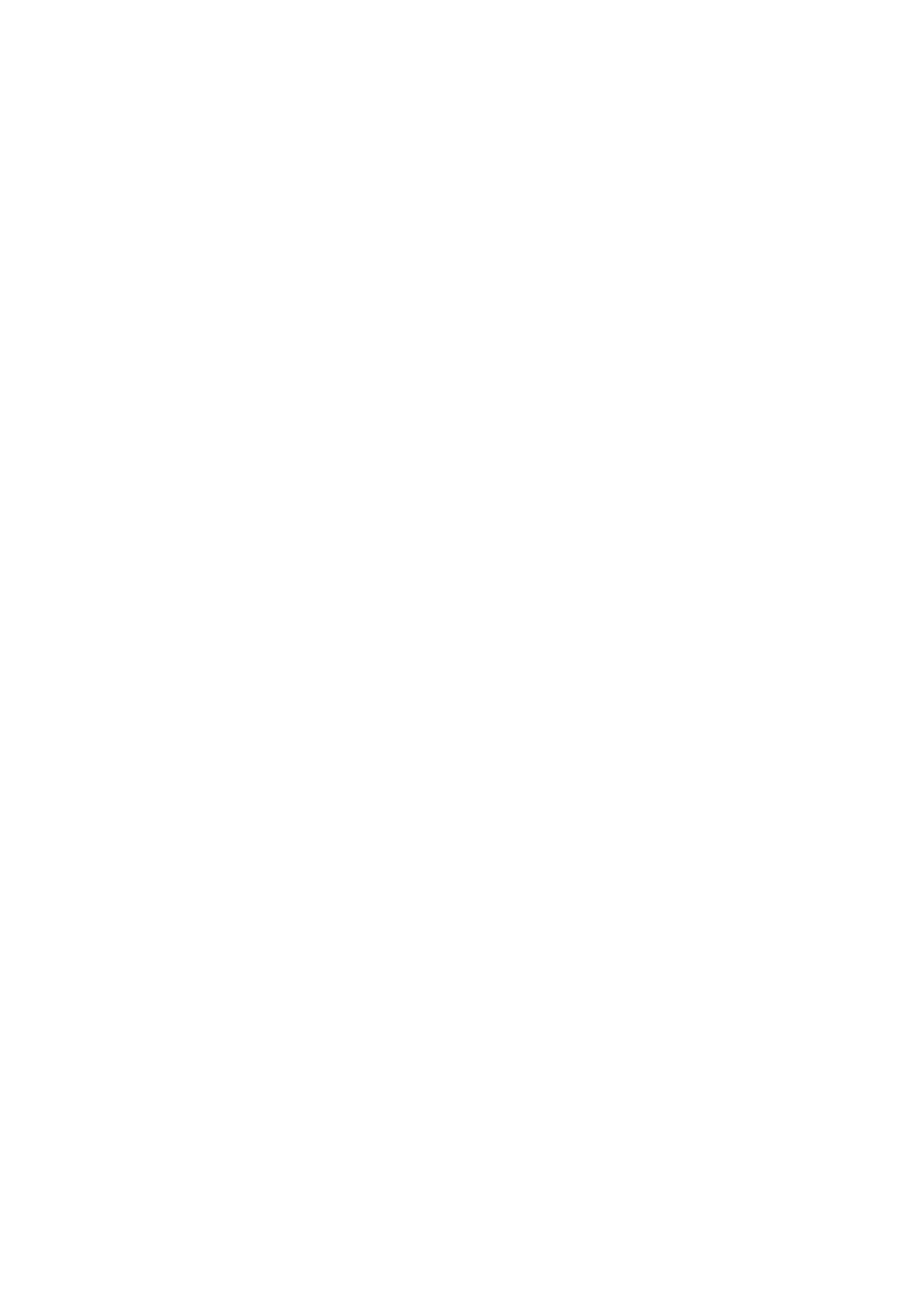

Supplement: Supplementary file 6 [file Presentation_1.PPTX]
