# Supplementary figures and images for: A Probiotic Mixture Induces Anxiolytic- and Antidepressive-Like Effects in Fischer and Maternally Deprived Long Evans Rats
Source: Front Behav Neurosci. 2020 Nov 12;14:581296. doi: 10.3389/fnbeh.2020.581296 (PMC7708897; doi:10.3389/fnbeh.2020.581296)

## Slide 1
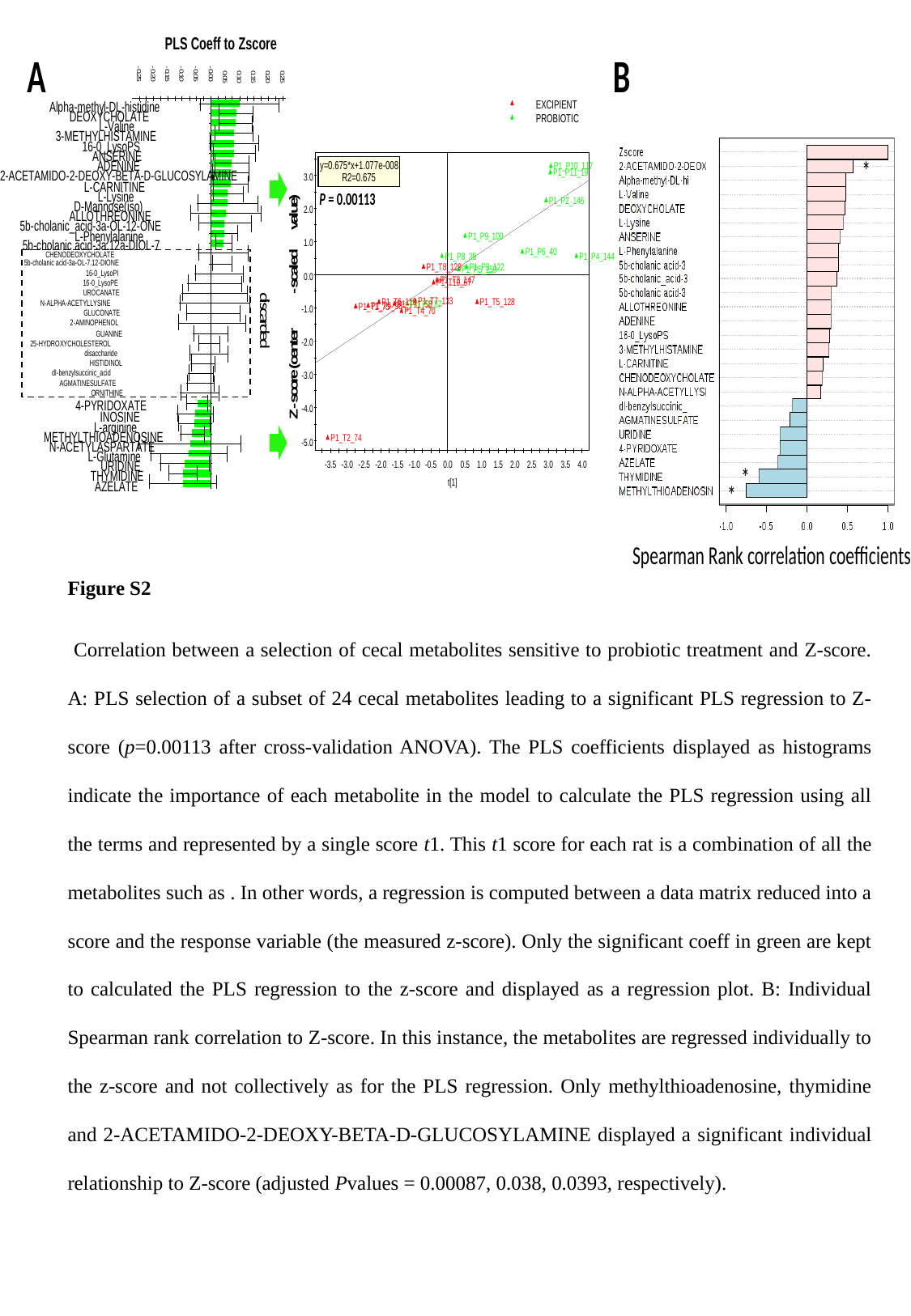

Supplement: Supplementary file 7 [file Presentation_2.PPTX]
