# Supplementary material for: A Probiotic Mixture Induces Anxiolytic- and Antidepressive-Like Effects in Fischer and Maternally Deprived Long Evans Rats
Source: Front Behav Neurosci. 2020 Nov 12;14:581296. doi: 10.3389/fnbeh.2020.581296 (PMC7708897; doi:10.3389/fnbeh.2020.581296)
Supplement: Supplementary file 8 [file Presentation_3.PPTX]

## Slide 1
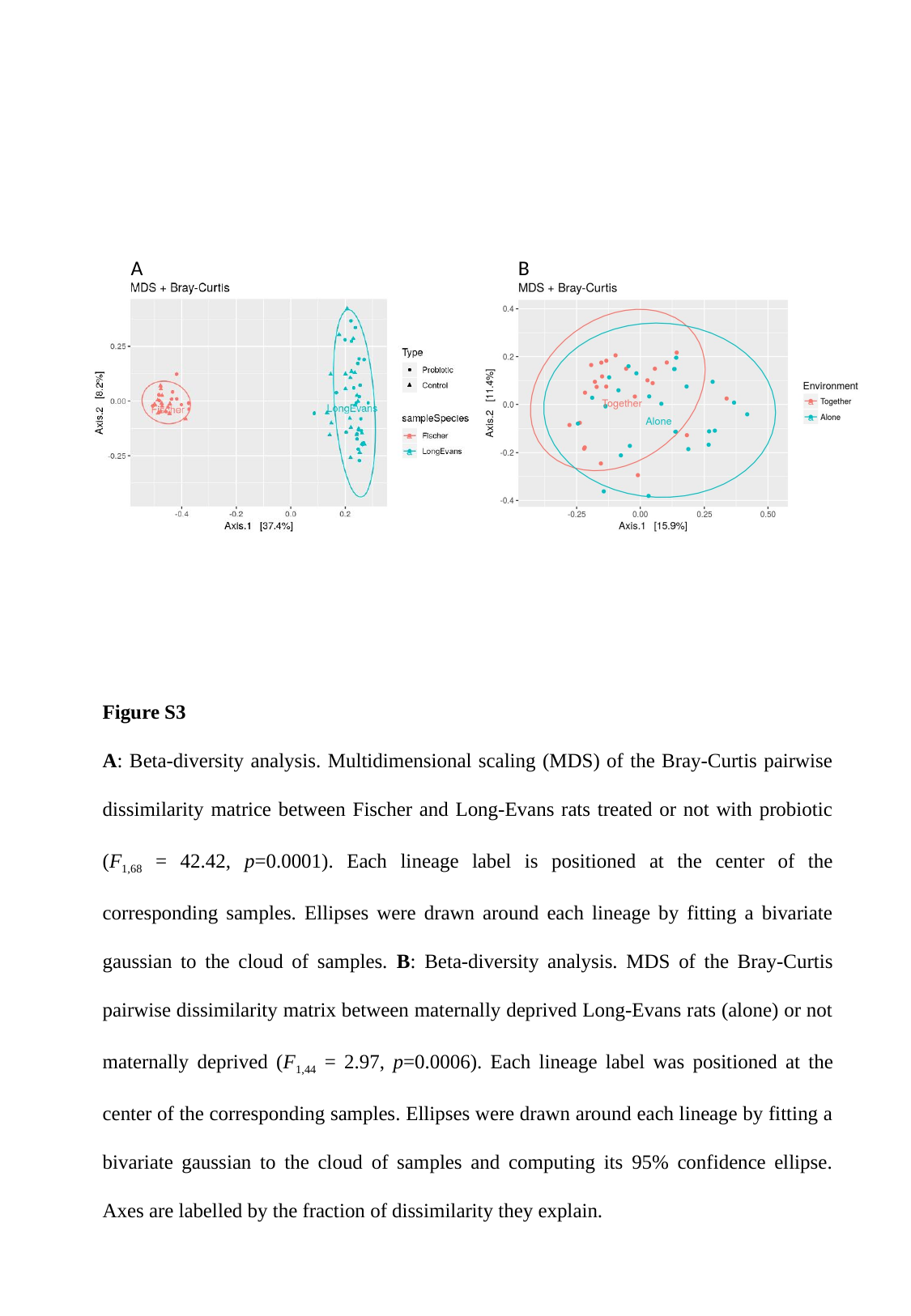

A
B
Figure S3
A: Βeta-diversity analysis. Multidimensional scaling (MDS) of the Bray-Curtis pairwise dissimilarity matrice between Fischer and Long-Evans rats treated or not with probiotic (F1,68 = 42.42, p=0.0001). Each lineage label is positioned at the center of the corresponding samples. Ellipses were drawn around each lineage by fitting a bivariate gaussian to the cloud of samples. B: Beta-diversity analysis. MDS of the Bray-Curtis pairwise dissimilarity matrix between maternally deprived Long-Evans rats (alone) or not maternally deprived (F1,44 = 2.97, p=0.0006). Each lineage label was positioned at the center of the corresponding samples. Ellipses were drawn around each lineage by fitting a bivariate gaussian to the cloud of samples and computing its 95% confidence ellipse. Axes are labelled by the fraction of dissimilarity they explain.
