# Supplementary material for: A Probiotic Mixture Induces Anxiolytic- and Antidepressive-Like Effects in Fischer and Maternally Deprived Long Evans Rats
Source: Front Behav Neurosci. 2020 Nov 12;14:581296. doi: 10.3389/fnbeh.2020.581296 (PMC7708897; doi:10.3389/fnbeh.2020.581296)
Supplement: Supplementary file 9 [file Presentation_4.PPTX]

## Slide 1
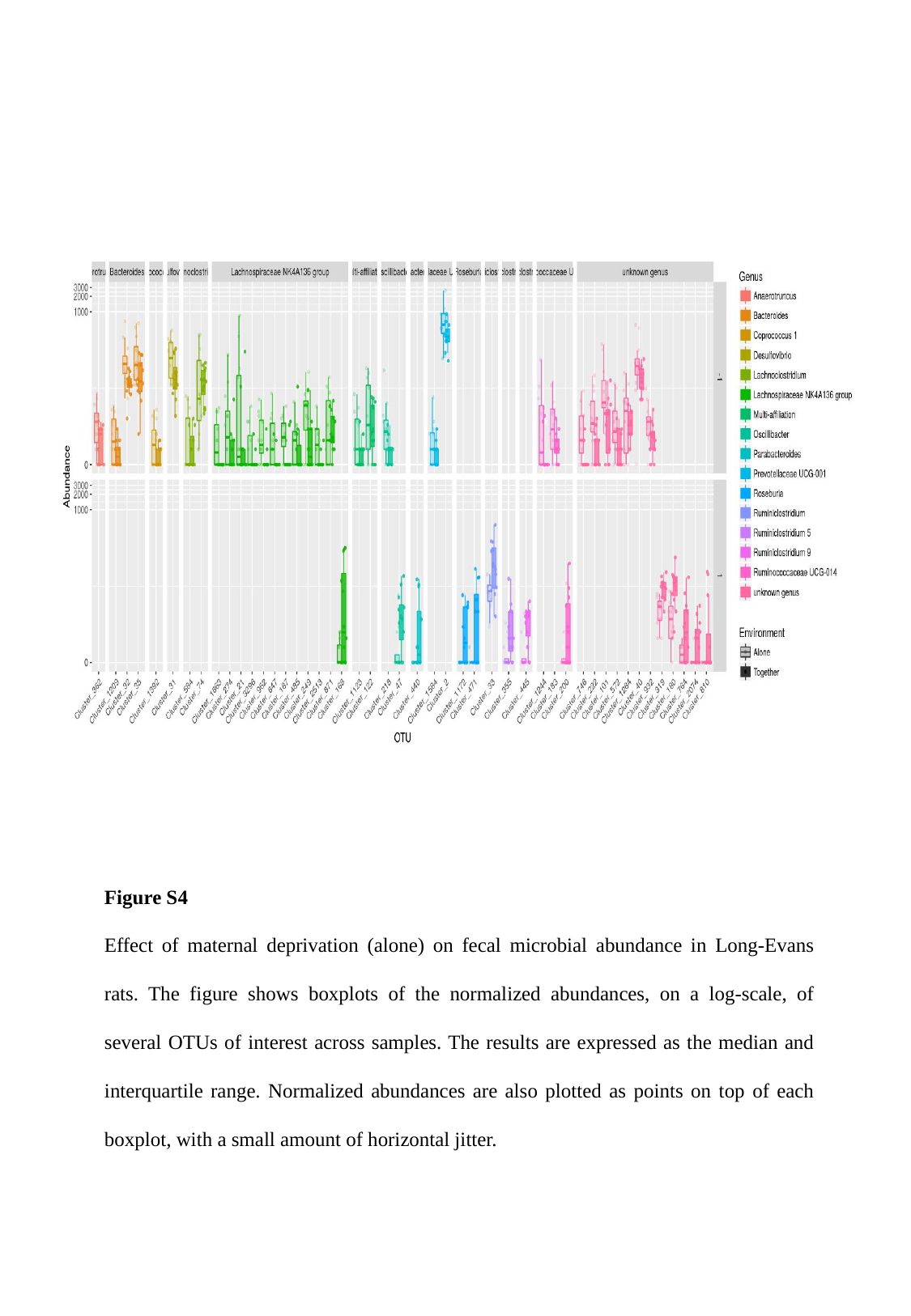

Figure S4
Effect of maternal deprivation (alone) on fecal microbial abundance in Long-Evans rats. The figure shows boxplots of the normalized abundances, on a log-scale, of several OTUs of interest across samples. The results are expressed as the median and interquartile range. Normalized abundances are also plotted as points on top of each boxplot, with a small amount of horizontal jitter.
